# Event-Driven Intensification and Persistent Therapeutic Inertia in LDL-C Management Among 1,033,703 Patients With ASCVD

**DOI:** 10.1016/j.jacadv.2026.103160

**Published:** 2026-08-22

**Authors:** Satoshi Shoji, Peter Shrader, Nikki L.B. Freeman, Nishant P. Shah, Vera Bittner, Tanya Wilcox, Amanda C. Doran, Cezary Wójcik, Ran Jin, Laney K. Jones, Eric D. Peterson, Ann Marie Navar

**Affiliations:** a Duke Clinical Research Institute, Durham, North Carolina, USA; b Division of Cardiology and Department of Medicine, Duke University School of Medicine, Durham, North Carolina, USA; c Department of Cardiology, Keio University School of Medicine, Tokyo, Japan; d Department of Medicine, Division of Cardiovascular Disease, University of Alabama at Birmingham, Birmingham, Alabama, USA; e University of Utah Health, Salt Lake City, Utah, USA; f Department of Medicine, Division of Cardiovascular Medicine, Vanderbilt University Medical Center, Nashville, Tennessee, USA; g Amgen Inc, Thousand Oaks, California, USA; h UT Southwestern Medical Center, Department of Medicine, Division of Cardiology, Dallas, Texas, USA

**Keywords:** atherosclerotic cardiovascular disease, lipid-lowering therapy, longitudinal trend, low-density lipoprotein cholesterol, recurrent event, therapeutic inertia

## Abstract

**Background:**

Long-term control of low-density lipoprotein cholesterol (LDL-C) is key to reducing the risk of atherosclerotic cardiovascular disease (ASCVD), yet most large-scale evaluations have been cross-sectional.

**Objectives:**

This study aimed to characterize the long-term trends in LDL-C among patients with ASCVD and to identify factors associated with reaching LDL-C goals.

**Methods:**

Using data from the cvMOBIUS-2 (Cardiovascular Multicenter Observational Investigation of Lipid Care in the United States-2) registry, which pools electronic health record data from 17 U.S. health systems, we identified adults with ASCVD and a baseline LDL-C ≥70 mg/dL. We used generalized estimating equations to model LDL-C trends and multivariable logistic regression to identify clinical factors associated with: 1) achieving an LDL-C <70 mg/dL at 12 months; and 2) therapeutic intensification, defined as statin initiation/increase or nonstatin addition, within 1 year.

**Results:**

Among 1,033,703 patients (median age 67.9 years; 49.2% women) with a median follow-up of 2.2 years (IQR: 1.1-3.8 years), LDL-C demonstrated a “drop-and-plateau” pattern: following an initial decline, mean LDL-C stalled well above 80 mg/dL and remained elevated throughout the remaining follow-up. A recurrent ASCVD event was strongly associated with both achieving LDL-C <70 mg/dL (OR: 2.18; 95% CI: 1.98-2.40) and therapeutic intensification (OR: 2.77; 95% CI: 2.34-3.27). Conversely, peripheral artery disease was associated with lower odds of goal attainment (OR: 0.70; 95% CI: 0.68-0.72) and intensification (OR: 0.73; 95% CI: 0.70-0.75).

**Conclusions:**

In ASCVD patients, LDL-C levels plateau well above guideline targets following an initial decline. Because lipid management remains predominantly event-driven, proactive, system-level monitoring and timely intervention are urgently needed to reduce cumulative above-goal LDL-C exposure.

Sustained reduction of low-density lipoprotein cholesterol (LDL-C) to guideline-recommended targets is a cornerstone of secondary prevention for atherosclerotic cardiovascular disease (ASCVD). Recent paradigms, including the updated 2026 dyslipidemia guidelines, advocate for increasingly aggressive targets (Class 1 recommendation for LDL-C <55 mg/dL in very high-risk ASCVD; Class 2a for not very high-risk). Furthermore, the 2025 European Society of Cardiology guidelines strongly endorse prompt intensification of lipid-lowering therapy (LLT) (Class 1) and upfront combination therapies (Class 2a) following an acute event.^1^^,^^2^ Despite these evolving, stringent targets and the availability of multiple effective therapies, cross-sectional studies consistently demonstrate profound gaps in care, with a vast majority of patients failing to achieve even the more conservative historical threshold of <70 mg/dL.^3^, ^4^, ^5^, ^6^, ^7^ Goal attainment is further limited by high rates of nonadherence and frequent discontinuation of statin and nonstatin therapy.^8^

However, most evidence evaluating real-world lipid management relies on cross-sectional snapshots, which fail to capture the dynamic nature of treatment over time. While a few studies provide a longitudinal perspective, they are often limited in scope. For example, the GOULD (Getting to an Improved Understanding of Low-Density Lipoprotein Cholesterol and Dyslipidemia Management) registry reported LDL-C goal attainment over 2 years among 5,006 patients; however, as a prospectively enrolled observational cohort, this selected population likely overestimates lipid control compared to routine practice.^9^^,^^10^ Consequently, true longitudinal LDL-C trajectories over multiple years remain poorly characterized across the broader ASCVD population.

The present study uses data from a diverse, multicenter U.S. registry of over 1 million adults with ASCVD and elevated baseline LDL-C (≥70 mg/dL). By analyzing long-term LDL-C trajectories and LLT use over time, this study provides a comprehensive assessment of longitudinal, real-world lipid management. Furthermore, we sought to identify the clinical factors associated with therapeutic intensification and LDL-C goal attainment among a secondary prevention population in contemporary routine clinical practice (Central Illustration).

**Central Illustration:**
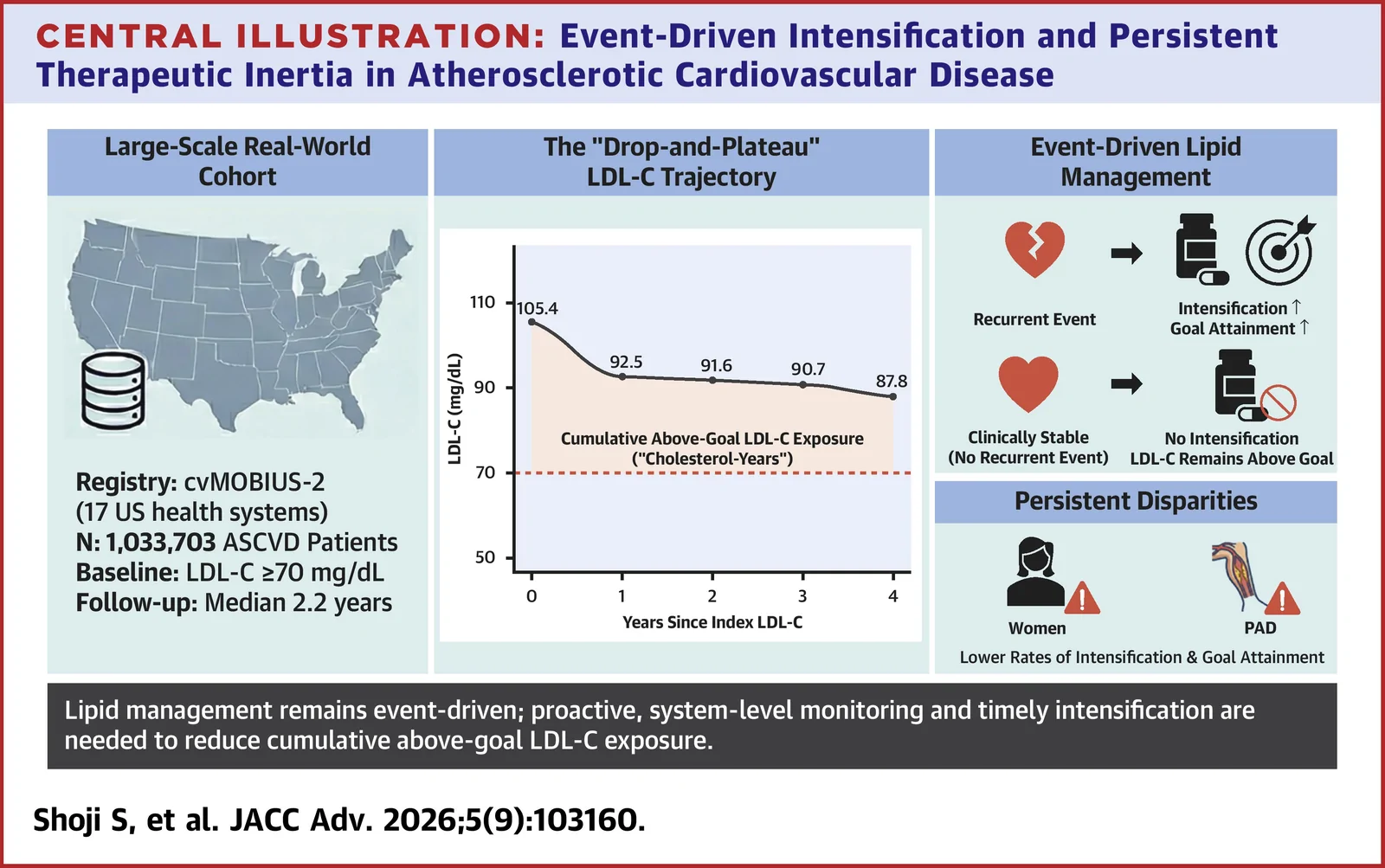
Event-Driven Intensification and Persistent Therapeutic Inertia in Atherosclerotic Cardiovascular Disease Among 1,033,703 patients with established atherosclerotic cardiovascular disease and baseline low-density lipoprotein cholesterol ≥70 mg/dL, mean low-density lipoprotein cholesterol levels rapidly plateaued well above the guideline-recommended goal of *<*70 mg/dL despite an initial decline, indicating cumulative above-goal low-density lipoprotein cholesterol exposure. A recurrent atherosclerotic cardiovascular disease event during follow-up was the factor most strongly associated with goal attainment and lipid-lowering therapy intensification, indicating that current lipid management is predominantly reactive rather than proactive. These findings highlight profound and persistent therapeutic inertia, emphasizing the urgent need for earlier, proactive treatment intensification to reduce cumulative above-goal low-density lipoprotein cholesterol exposure. cvMOBIUS-2 = Cardiovascular Multicenter Observational Investigation of Lipid Care in the United States-2; other abbreviations as in Figures 1 and 3.

## Methods

### Data source and study population

This study used data from the cvMOBIUS-2 (Cardiovascular Multicenter Observational Investigation of Lipid Care in the United States-2) registry.^4^^,^^11^ This registry contains longitudinal, de-identified electronic health record (EHR) data from 17 academic medical centers across the United States.^4^^,^^11^ The study cohort was derived from adult patients (≥18 years) with a documented diagnosis of ASCVD occurring between January 1, 2018, and June 26, 2025. The index date for each patient was defined as the date of their first LDL-C measurement following the first available ASCVD diagnosis. To be included in the study, this index LDL-C measurement was required to be ≥70 mg/dL and occur between January 1, 2019, and June 26, 2025. This ensured all patients had at least 1 year of data available to identify comorbidities, procedures, and prior cardiovascular events. Clinical ASCVD was defined based on EHR diagnosis codes for coronary artery disease (CAD), ischemic cerebrovascular disease (CeVD), or peripheral artery disease (PAD), or a history of coronary, peripheral, or carotid revascularization (Supplemental Appendix). Baseline comorbidities, including familial hypercholesterolemia, were identified using standard International Classification of Diseases-Tenth Revision diagnosis codes.

From this baseline population, 3 cohorts were created to evaluate the following: 1) longitudinal LDL-C trends; 2) 12-month LDL-C goal attainment; and 3) intensification of LLT. For the analysis of LDL-C trends, patients were required to have at least 1 follow-up LDL-C measurement after the index date. For the analysis of 12-month LDL-C goal attainment, we further subset the population to identify those with at least 1 LDL-C measurement between 6 and 18 months after the index date; the last available LDL-C measurement within this window was used to determine goal attainment. For the analysis of intensification of LLT, we identified any intensification events occurring within the first 12 months (365 days) following the index date, regardless of the presence or absence of any follow-up LDL-C levels. The analysis cohort for this outcome was limited to patients with a record of at least 1 prescription for any medication during this period, ensuring they were actively engaged with the health care system. Furthermore, we conducted a prespecified subgroup analysis of patients with “very high-risk ASCVD,” defined as having an established ASCVD diagnosis plus 2 or more high-risk conditions (age ≥65 years, hypertension, diabetes mellitus, heart failure, current smoking, or baseline LDL-C >100 mg/dL).

This project received approval from the Duke University Institutional Review Board, and each participating site also obtained approval from its local Institutional Review Board. A waiver of consent was obtained for the use of this deidentified, retrospectively collected EHR data.

### Statistical analysis

First, baseline demographic and clinical characteristics were summarized. We described the overall population with a baseline LDL-C ≥70 mg/dL, as well as the subcohort of patients with at least 1 LDL-C measurement at 1 year (based on LDL-C data between 6 and 18 months). For this subcohort, characteristics were further stratified by whether the 1-year LDL-C was <70 mg/dL or ≥70 mg/dL. Baseline demographic and clinical characteristics were summarized using descriptive statistics. Continuous variables were reported as medians with IQRs, and categorical variables were expressed as counts with percentages.

Subsequent analyses were designed to characterize: 1) longitudinal LDL-C trends; 2) identify factors associated with achieving an LDL-C <70 mg/dL at 1 year; and 3) identify factors associated with intensification of LLT within 1 year. Across all longitudinal tracking and outcome evaluations, all-cause mortality was treated as a censoring event; patients contributed data to the study and respective models until the date of death or the end of their specific follow-up period.

### Longitudinal trends in LDL-C levels

To evaluate population-level trends in LDL-C, we used generalized estimating equations (GEE) to model repeated, irregularly spaced LDL-C measurements over time. To flexibly capture the nonlinear pattern of LDL-C change, time since baseline was modeled using restricted cubic splines with 4 knots placed at the 5th, 35th, 65th, and 95th percentiles of the time distribution. The resulting mean LDL-C trends and 95% confidence bands were plotted for the overall population. For the GEE models mapping the longitudinal trajectory, we utilized an independent working correlation matrix and employed robust empirical standard errors to account for within-patient correlation over time. Hospital-level clustering was evaluated by including the health system as a random intercept in complementary mixed-effect models. The primary trajectory models evaluated unadjusted population-level trends over time to reflect real-world implementation gaps without masking effects through baseline covariate adjustment. To assess the potential impact of regression to the mean, we performed a sensitivity analysis restricted to a subcohort of patients who had at least 1 additionally documented elevated LDL-C (≥70 mg/dL) in the 12 months prior to their index measurement.

LDL-C trends were plotted stratified by patient sex and race to explore potential disparities. Furthermore, to visualize the relationship between an early recurrent clinical event and the long-term management pattern for discussion purposes, we performed a landmark-style analysis. In this descriptive analysis, patients were classified based on whether they experienced a recurrent ASCVD event within the first year, and their complete mean LDL-C trends were plotted from baseline.

For individual-level changes, we described transitions between 4 predefined LDL-C categories (≥100, 70-99, 55-69, and <55 mg/dL) across 3 specific follow-up windows: year 1: 1 to 13 months, year 2: 13 to 25 months, and year 3: 25 to 37 months post-baseline. These cohorts were not mutually exclusive, as patients could contribute measurements to multiple windows. This windowed approach, starting at 1-month post-baseline, was used to capture clinically relevant follow-up measurements after the index visit. For each time window, the last available LDL-C value recorded within the specific window was used. These transitions were illustrated using Sankey diagrams.

### Factors associated with 12-month LDL-C goal attainment

We used multivariable logistic regression with GEE to evaluate factors associated with achieving an LDL-C level <70 mg/dL at 12 months (6-18 months). Covariates for adjustment included age, sex, race, ethnicity, insurance type, baseline LDL-C (categorized as 70-99 vs ≥100 mg/dL), and key comorbidities present at baseline (systolic heart failure, diastolic heart failure, atrial fibrillation/flutter, and dialysis). Other covariates were defined and categorized as follows: body mass index (categorized as <25, 25-29.9, 30-39.9, and ≥40 kg/m^2^); type of ASCVD (CAD only [reference], PAD only, CeVD only, polyvascular disease, and PAD + CeVD); recent cardiovascular events (myocardial infarction, stroke, percutaneous coronary intervention, or coronary artery bypass graft) within 1 year before the index date; estimated glomerular filtration rate (no chronic kidney disease [reference], stage 2: 60 to 89, stage 3: 30 to 59, stage 4/5: <30 or on dialysis); and the clinical epoch of the index LDL-C (categorized as pre-COVID [2019], pandemic [2020], and post-pandemic [2021-2024]). A recurrent ASCVD event was included as a binary covariate, defined as the occurrence of one or more qualifying events (myocardial infarction, stroke, percutaneous coronary intervention, or coronary artery bypass graft) documented in the EHR using International Classification of Diseases diagnosis codes or Current Procedural Terminology procedure codes. To ensure strict temporal precedence, recurrent ASCVD was treated dynamically as a time-varying binary covariate. Because all patients had an established ASCVD diagnosis at baseline as an inclusion criterion, any qualifying event occurring after the index date was considered recurrent. For each patient, this covariate transitioned from 0 to 1 exclusively at the time point immediately following their first post-index event. We utilized this binary transition rather than a cumulative categorical count due to the statistical sparsity of multiple independent recurrent events occurring within the brief window prior to therapeutic intensification.

### Factors associated with intensification of LLT

We also conducted multivariable logistic regression with GEE to evaluate factors associated with therapeutic intensification within 1 year of the index date. Intensification was defined as either statin initiation or an increase in statin dose/intensity, or the addition of a nonstatin therapy (ezetimibe, proprotein convertase subtilisin/kexin type 9 inhibitor [PCSK9 inhibitor], inclisiran, bempedoic acid, or bile acid sequestrants). This model included the same set of covariates as the model for 12-month LDL-C goal attainment. Similarly, to avoid temporal bias, the recurrent ASCVD event covariate was handled dynamically; for each patient, an event was only considered a predictor if it occurred before the date of therapeutic intensification. For this analysis, prespecified subgroup analyses were conducted, stratifying the results by: 1) type of ASCVD; and 2) the calendar year of the index LDL-C measurement.

### Sensitivity and bias analyses

To assess potential informative observation and selection biases, we performed 2 sensitivity analyses. First, within the longitudinal trajectory cohort (N = 671,353), we compared baseline characteristics between patients with frequent (≥1 LDL-C test/year) vs infrequent (<1 LDL-C test/year) follow-up measurements. Second, within the overall baseline cohort (N = 1,033,703), we compared characteristics between those included vs excluded from the 12-month goal attainment analysis to evaluate potential selection bias within this specific subcohort.

Any missing data for categorical covariates were included as a separate category in the analysis. Statistical significance was established based on a 2-sided *P* value of <0.05, without adjusting for multiple comparisons. All statistical analyses were performed using SAS software (version 9.4; SAS Institute).

## Results

### Study population

The initial data set included 2,628,239 adult patients with a documented diagnosis of ASCVD. Of these, 1,626,683 had a qualifying LDL-C measurement after their diagnosis and within the specified study window. We then restricted the cohort to those with a baseline LDL-C level ≥70 mg/dL, which resulted in a final baseline population of 1,033,703 patients. From this population, the specific analytical cohorts were derived: 671,353 patients were included in the LDL-C trend analysis, 322,820 in the 12-month goal attainment analysis, and 712,057 in the intensification analysis.

Baseline characteristics are detailed in Table 1. The overall cohort with a baseline LDL-C ≥70 mg/dL (N = 1,033,703) had a median age of 67.9 years, and 49.2% were female. The baseline ASCVD subtypes comprised CAD only (49.0%), CeVD only (20.2%), and PAD only (13.6%), with 17.3% of patients having polyvascular disease (including 2.1% with PAD + CeVD). The median baseline LDL-C for this group was 99.0 mg/dL (IQR: 83.0-124.0 mg/dL). Of note, among the 22,857 patients with extreme hypercholesterolemia (baseline LDL-C ≥190 mg/dL), 3.7% had a formal International Classification of Diseases diagnosis of familial hypercholesterolemia.

**Table 1:** Baseline Characteristics of all Patients With Baseline LDL-C ≥70 mg/dL and of the 1-Year Follow-Up Cohort, Stratified by LDL-C Goal Attainment

	All Patients with LDL-C >70 mg/dL at Baseline (N = 1,033,703)	Those With at Least 1 LDL-C Measurement in 1 Year (N = 322,820)	1 Year LDL-C <70 mg/dL (N = 74,409)	1 Year LDL-C ≥70 mg/dL (N = 248,411)
Demographics				
Age at index date, y	67.9 (59.4, 75.7)	68.8 (61.1, 75.8)	68.8 (61.2, 75.6)	68.9 (61.1, 75.8)
Female	508,118 (49.2%)	158,522 (49.2%)	29,460 (39.6%)	129,062 (52.0%)
Race				
American Indian/Alaska Native	2,863 (0.3%)	728 (0.2%)	166 (0.2%)	562 (0.2%)
Asian	16,385 (1.6%)	4,966 (1.5%)	1,313 (1.8%)	3,653 (1.5%)
Black	163,848 (15.9%)	46,263 (14.3%)	8,854 (11.9%)	37,409 (15.1%)
White	803,759 (77.8%)	259,401 (80.4%)	61,206 (82.3%)	198,195 (79.8%)
Other	3,824 (0.4%)	936 (0.3%)	213 (0.3%)	723 (0.3%)
Unknown/missing	43,023 (4.2%)	10,526 (3.3%)	2,657 (3.6%)	7,869 (3.2%)
Hispanic ethnicity	47,301 (5.0%)	12,903 (4.3%)	3,346 (4.9%)	9,557 (4.2%)
Insurance				
Medicare	477,419 (46.8%)	161,396 (50.7%)	36,078 (49.3%)	125,318 (51.2%)
Medicaid	42,531 (4.2%)	9,386 (2.9%)	2,219 (3.0%)	7,167 (2.9%)
Government	15,965 (1.6%)	3,650 (1.1%)	861 (1.2%)	2,789 (1.1%)
Private	241,618 (23.7%)	76,579 (24.1%)	17,238 (23.5%)	59,341 (24.2%)
Other	121,952 (12.0%)	35,747 (11.2%)	9,175 (12.5%)	26,572 (10.8%)
None/missing	119,877 (11.8%)	31,439 (9.9%)	7,631 (10.4%)	23,808 (9.7%)
Type of ASCVD				
CAD only	506,467 (49.0%)	162,509 (50.3%)	40,619 (54.6%)	121,890 (49.1%)
PAD only	140,109 (13.6%)	51,954 (16.1%)	8,036 (10.8%)	43,918 (17.7%)
CeVD only	208,590 (20.2%)	55,971 (17.3%)	11,994 (16.1%)	43,977 (17.7%)
Polyvascular disease	156,622 (15.2%)	45,824 (14.2%)	12,247 (16.5%)	33,577 (13.5%)
PAD + CeVD	21,915 (2.1%)	6,562 (2.0%)	1,513 (2.0%)	5,049 (2.0%)
Comorbidities				
Dialysis	15,569 (1.5%)	4,824 (1.5%)	1,494 (2.0%)	3,330 (1.3%)
Current smoking	128,827 (12.5%)	34,898 (10.8%)	8,398 (11.3%)	26,500 (10.7%)
Atrial fibrillation or flutter	142,706 (13.8%)	50,811 (15.7%)	13,455 (18.1%)	37,356 (15.0%)
Familial hypercholesterolemia	5,574 (0.5%)	2,669 (0.8%)	571 (0.8%)	2098 (0.8%)
Liver disease	70,154 (6.8%)	27,009 (8.4%)	6,508 (8.7%)	20,501 (8.3%)
Systolic HF	81,545 (7.9%)	26,814 (8.3%)	8,040 (10.8%)	18,774 (7.6%)
Diastolic HF	67,396 (6.5%)	23,617 (7.3%)	6,394 (8.6%)	17,223 (6.9%)
Hypertension	602,567 (58.3%)	222,683 (69.0%)	53,358 (71.7%)	169,325 (68.2%)
HIV	5,095 (0.5%)	1,911 (0.6%)	419 (0.6%)	1,492 (0.6%)
Psoriasis	14,218 (1.4%)	5,960 (1.8%)	1,402 (1.9%)	4,558 (1.8%)
Rheumatoid arthritis	21,356 (2.1%)	7,800 (2.4%)	1,775 (2.4%)	6,025 (2.4%)
Events in the past 1 year				
MI hospitalization	61,573 (6.0%)	12,458 (3.9%)	5,584 (7.5%)	6,874 (2.8%)
Stroke hospitalization	70,403 (6.8%)	9,994 (3.1%)	4,016 (5.4%)	5,978 (2.4%)
CABG	8,188 (0.8%)	2,128 (0.7%)	781 (1.0%)	1,347 (0.5%)
PCI	28,867 (2.8%)	7,028 (2.2%)	3,238 (4.4%)	3,790 (1.5%)
Vitals and labs				
BMI, kg/m^2^	29.0 (25.4, 33.8)	29.3 (25.8, 34.0)	29.5 (26.0, 34.0)	29.3 (25.8, 33.9)
LDL-C, mg/dL	99.0 (83.0, 124.0)	98.0 (82.0, 122.0)	86.0 (76.0, 107.0)	101.0 (85.0, 125.0)
HDL-C, mg/dL	48.0 (39.0, 60.0)	49.0 (40.0, 60.0)	46.0 (38.0, 58.0)	49.0 (41.0, 61.0)
Non-HDL-C, mg/dL	125.0 (105.0, 154.0)	124.0 (104.0, 152.0)	112.0 (96.0, 139.0)	127.0 (107.0, 155.0)
Triglycerides, mg/dL	116.0 (84.0, 166.0)	118.0 (85.0, 167.0)	119.0 (85.0, 171.0)	117.0 (85.0, 166.0)
Total cholesterol, mg/dL	177.0 (154.0, 206.0)	176.0 (154.0, 206.0)	163.0 (144.0, 190.0)	180.0 (157.0, 210.0)
HbA1c, %	5.9 (5.5, 6.8)	6.0 (5.6, 6.9)	6.1 (5.6, 7.2)	6.0 (5.6, 6.9)
eGFR, mL/min/1.73 m^2^	76.0 (60.0, 91.0)	76.0 (60.0, 90.0)	75.0 (60.0, 90.0)	76.0 (61.0, 90.0)
Recurrent ASCVD events during follow-up				
Any	-	19,307 (6.0%)	8,279 (11.1%)	11,028 (4.4%)
MI	-	6,449 (2.0%)	2,724 (3.7%)	3,725 (1.5%)
Stroke	-	6,827 (2.1%)	2,723 (3.7%)	4,104 (1.7%)
PCI	-	5,506 (1.7%)	2,564 (3.4%)	2,942 (1.2%)
CABG	-	4,031 (1.2%)	1,886 (2.5%)	2,145 (0.9%)

Values are median (IQR) or n (%). The table displays 4 patient groups: 1) all patients with a baseline LDL-C ≥70 mg/dL (N = 1,033,703); 2) the cohort with at least one LDL-C measurement at 1 year (6-18 months post-baseline) (N = 322,820); this cohort is further stratified into; 3) those who achieved an LDL-C <70 mg/dL at 1 year (N = 74,409); and 4) those who did not (LDL-C ≥70 mg/dL) (N = 248,411).

ASCVD = atherosclerotic cardiovascular disease; BMI = body mass index; CABG = coronary artery bypass grafting; CAD = coronary artery disease; CeVD = cerebrovascular disease; eGFR = estimated glomerular filtration rate; HbA1c = glycated hemoglobin; HDL-C = high-density lipoprotein cholesterol; HF = heart failure; LDL-C = low-density lipoprotein cholesterol; MI = myocardial infarction; PAD = peripheral artery disease; PCI = percutaneous coronary intervention.

The cohort included in the 12-month goal attainment analysis (N = 322,820) was broadly similar, with a median age of 68.8 years and a median baseline LDL-C of 98.0 mg/dL (IQR: 82.0-122.0 mg/dL). Within this group, 74,409 patients (23.0%) achieved the goal of an LDL-C <70 mg/dL at 1 year. Notably, patients who achieved the goal had a lower median baseline LDL-C compared with those who did not (86.0 mg/dL vs 101.0 mg/dL, respectively). Furthermore, a smaller proportion of patients who achieved the goal were female compared with those who did not (39.6% vs 52.0%).

### Longitudinal LDL-C trends

**Figure 1:**
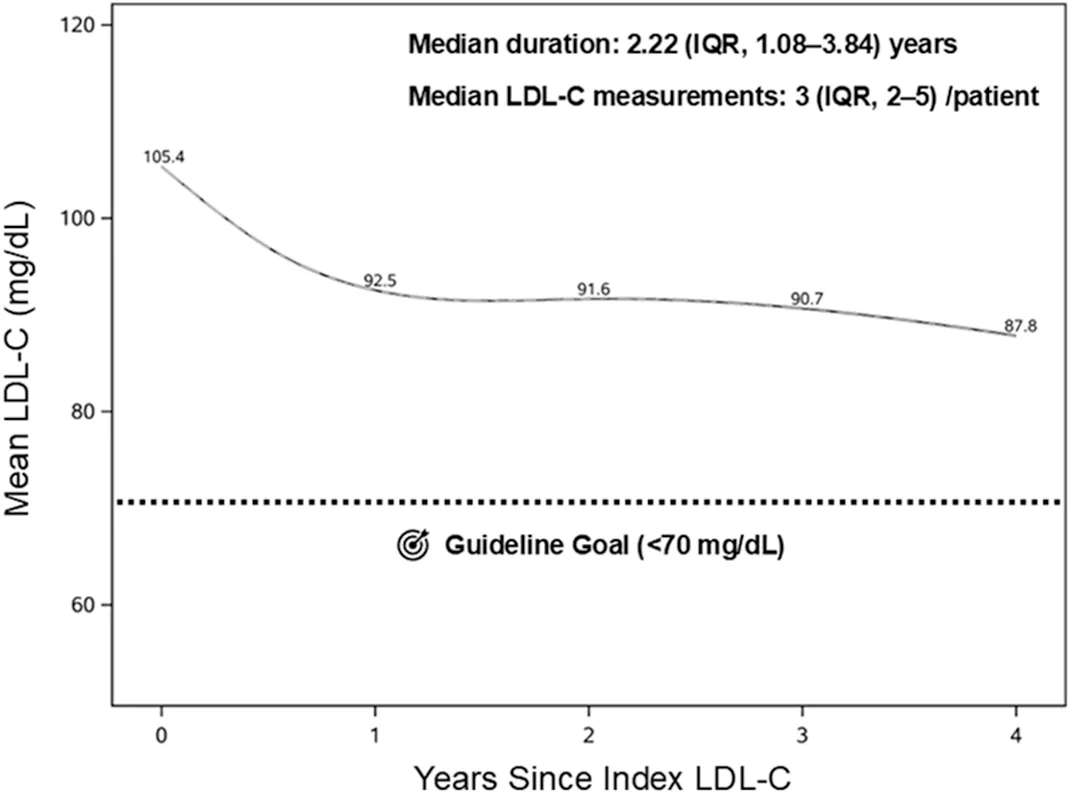
Mean Longitudinal Low-Density Lipoprotein Cholesterol Trajectory in Patients With Atherosclerotic Cardiovascular Disease and Baseline Low-Density Lipoprotein Cholesterol ≥70 mg/dL The solid line represents the estimated mean low-density lipoprotein cholesterol trajectory derived from a generalized estimating equation model. This model utilized restricted cubic splines to flexibly capture the nonlinear relationship between time since baseline and low-density lipoprotein cholesterol levels; the shaded bands represent the 95% CIs. The dashed horizontal line indicates the guideline-recommended threshold of <70 mg/dL. The analysis included 671,353 patients (median follow-up: 2.22 [IQR: 1.08-3.84] years; median number of low-density lipoprotein cholesterol measurements: 3 [IQR: 2-5], including the index measurement). LDL-C = low-density lipoprotein cholesterol.

The population-level trend analysis included 671,353 patients with a median follow-up of 2.22 (IQR: 1.08-3.84) years. Patients had a median of 3 (IQR: 2-5) LDL-C measurements, including the index value. The GEE model revealed a distinct nonlinear pattern (Figure 1). Mean LDL-C declined from a baseline of approximately 105 mg/dL to approximately 92 mg/dL over the first year. Following this initial decline, the curve flattened, forming a prolonged plateau well above the 70 mg/dL target. Even after 4 years of follow-up, the mean LDL-C remained substantially above the guideline-recommended threshold. In the sensitivity analysis restricted to patients with historically sustained LDL-C elevations, the longitudinal trajectory mirrored the primary analysis (Supplemental Figure 1). While absolute mean LDL-C levels were marginally higher at each time point, the characteristic “drop-and-plateau” pattern remained robust, confirming that the observed initial decline is not primarily driven by statistical regression to the mean. Further stratified analyses demonstrated persistent differences in LDL-C trends by sex and race (Supplemental Figures 2 and 3), with female patients and Black patients demonstrating consistently higher mean LDL-C levels compared to their male and White/Asian counterparts, respectively.

**Figure 2:**
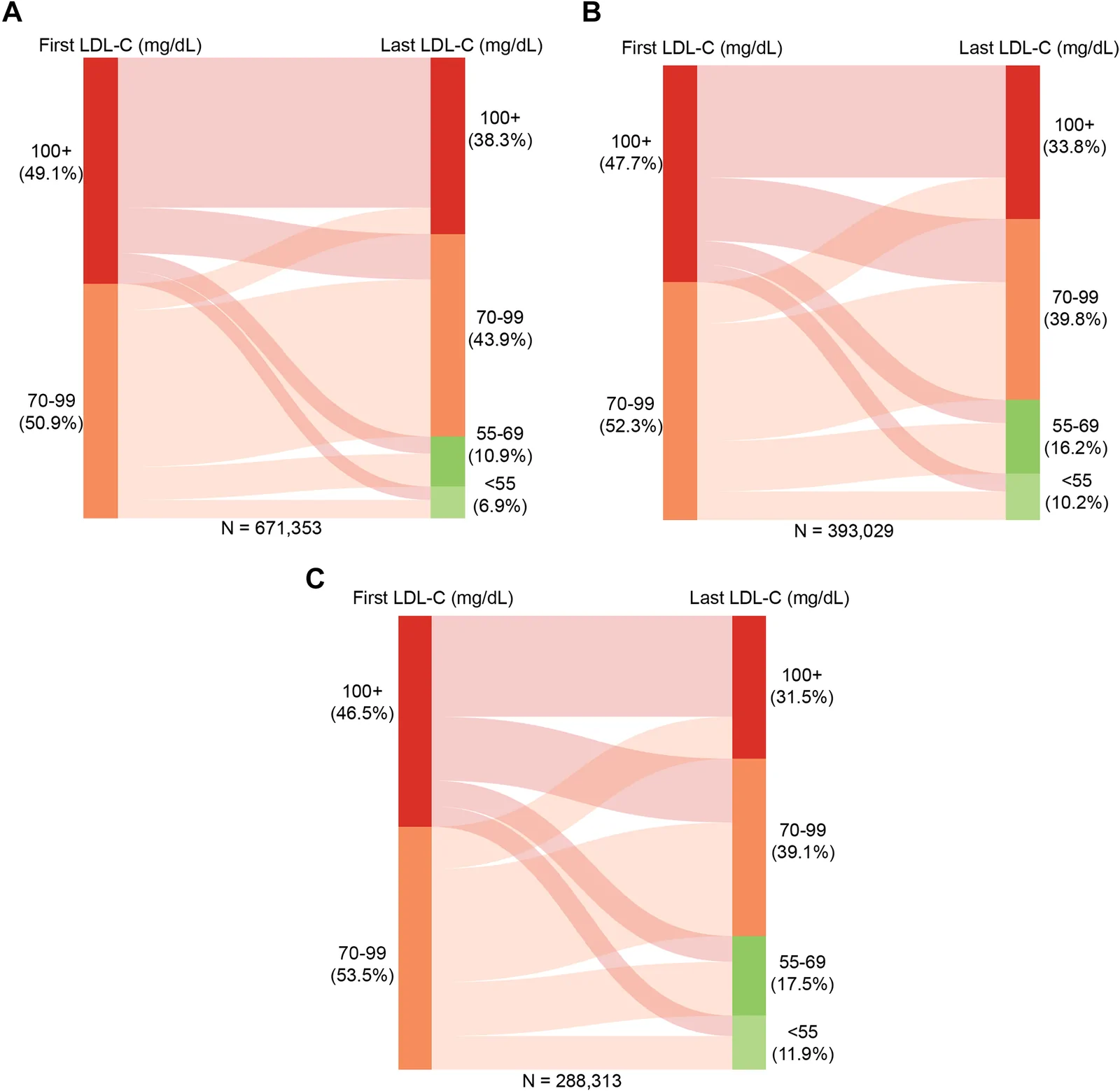
Sankey Diagrams of Patient Transitions Between Low-Density Lipoprotein Cholesterol Categories Over Time (A) Transitions from baseline to 1 year (1-13 months), (B) Transitions from baseline to 2 years (13-25 months), and (C) Transitions from baseline to 3 years (25-37 months). The diagrams illustrate the flow of patients from their baseline low-density lipoprotein cholesterol categories (left bars; stratified into 70-99 mg/dL and ≥100 mg/dL) to their low-density lipoprotein cholesterol categories at follow-up (right bars). The height of each bar segment represents the percentage of patients within that category at that time point. The flow paths show the proportion of patients transitioning between categories. For each follow-up window, the last available low-density lipoprotein cholesterol measurement was used. The percentages for the baseline low-density lipoprotein cholesterol categories differ slightly across panels (A), (B), and (C) because each panel's denominator is restricted to only those patients who had a follow-up low-density lipoprotein cholesterol measurement within the specified time window. Abbreviation as in Figure 1.

Visualization of individual-level transitions using Sankey diagrams illustrated a profound and persistent failure to achieve guideline-recommended goals (Figure 2). The proportion of patients who had not achieved an LDL-C <70 mg/dL was 82.2% at 1 year, 73.6% at 2 years, and 70.6% at 3 years. The gap in care was even more pronounced for the more aggressive target of <55 mg/dL, with failure rates of 93.1%, 89.8%, and 88.1% at 1, 2, and 3 years, respectively. Finally, when evaluating the last available measurement for each patient across their entire follow-up period—which extended beyond the 3-year windows—68.8% of patients ultimately did not achieve a final LDL-C <70 mg/dL, and 86.5% did not achieve an LDL-C <55 mg/dL.

### Factors associated with achieving LDL-C <70 mg/dL

**Figure 3:**
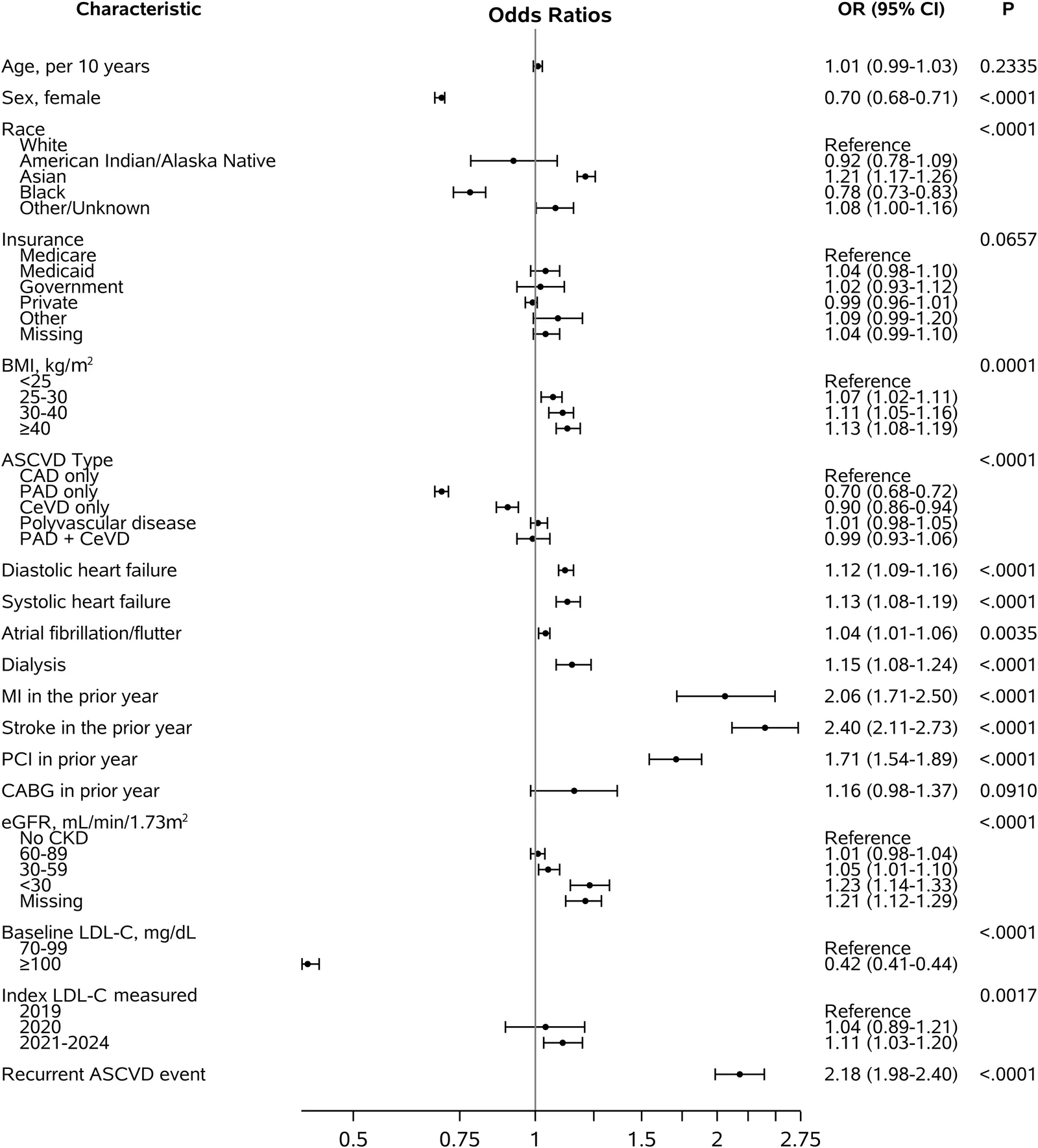
Adjusted ORs for Achieving Low-Density Lipoprotein Cholesterol <70 mg/dL at 12 Months The forest plot displays the adjusted ORs and 95% CIs from the multivariable logistic regression model for the outcome of achieving a low-density lipoprotein cholesterol level <70 mg/dL at 12 months (6-18 months post-baseline). An OR > 1 indicates higher odds of achieving the goal, while an OR < 1 indicates lower odds. The analysis included 322,820 patients. ASCVD = atherosclerotic cardiovascular disease; BMI = body mass index; CABG = coronary artery bypass grafting; CAD = coronary artery disease; CeVD = cerebrovascular disease; CKD = chronic kidney disease; eGFR = estimated glomerular filtration rate; LDL-C = low-density lipoprotein cholesterol; MI = myocardial infarction; PAD = peripheral artery disease; PCI = percutaneous coronary intervention; other abbreviation as in Figure 1.

Among 322,820 individuals with a follow-up LDL-C level around 1 year, 23.0% achieved an LDL-C <70 mg/dL at 1 year. In the multivariable adjusted analysis (Figure 3), several patient characteristics were associated with lower odds of achieving an LDL-C <70 mg/dL at 12 months. These included female sex (OR: 0.70; 95% CI: 0.68-0.71), Black race (OR: 0.78; 95% CI: 0.73-0.83), and having PAD only (OR: 0.70; 95% CI: 0.68-0.72) or CeVD only (OR: 0.90; 95% CI: 0.86-0.94) compared with CAD only. The PAD + CeVD subgroup did not show a statistically significant association, though estimates in this small subphenotype were imprecise due to wide CIs (OR: 0.99; 95% CI: 0.93-1.06). Conversely, several factors were associated with higher odds of achieving the goal. These included Asian race (OR: 1.21; 95% CI: 1.17-1.26), as well as having a recent clinical event in the year prior to the index date, such as a stroke (OR: 2.40; 95% CI: 2.11-2.73), myocardial infarction (OR: 2.06; 95% CI: 1.71-2.50), or percutaneous coronary intervention (OR: 1.71; 95% CI: 1.54-1.89). Among 322,820 individuals in this analysis, 19,307 (6.0%) had a recurrent ASCVD event, including 6,449 (2.0%) with a myocardial infarction, 6,827 (2.1%) with a stroke, 5,506 (1.7%) with percutaneous coronary intervention, and 4,031 (1.2%) with coronary artery bypass graft (not mutually exclusive). Notably, the occurrence of a recurrent ASCVD event during follow-up was strongly associated with achieving an LDL-C <70 mg/dL (OR: 2.18; 95% CI: 1.98-2.40). As expected, a baseline LDL-C ≥100 mg/dL was associated with significantly lower odds of achieving the goal compared to a baseline of 70 to 99 mg/dL (OR: 0.42; 95% CI: 0.41-0.44).

### Patterns of therapeutic intensification

Overall, among 712,057 patients with a baseline LDL-C ≥70 mg/dL, 26.9% underwent any intensification of LLT within 1 year (Table 2). This was most commonly accomplished by initiating or increasing the dose/intensity of statin therapy (22.8% of patients). The initiation of any nonstatin therapy was less common, occurring in 5.7% of patients overall. Among nonstatin therapies, ezetimibe was the most frequently initiated (4.1%), followed by PCSK9 inhibitors (1.4%).

**Table 2:** Rates of Therapeutic Intensification Within 1 Year, Stratified by Type of ASCVD and Recurrent Event Status

	Overall (N = 712,057)	CAD Only (n = 263,618)	PAD Only (n = 62,441)	CeVD Only (n = 86,978)	Polyvascular Disease (n = 266,891)	PAD + CeVD (n = 32,129)	Recurrent ASCVD Event (−) (n = 622,065)	Recurrent ASCVD Event (+) (n = 89,992)
Any therapeutic intensification	191,698 (26.9%)	71,540 (27.1%)	11,185 (17.9%)	21,517 (24.7%)	79,105 (29.6%)	8,351 (26.0%)	156,369 (25.1%)	35,329 (39.3%)
Initiate statin or dose increase	162,214 (22.8%)	58,318 (22.1%)	10,154 (16.3%)	19,773 (22.7%)	66,406 (24.9%)	7,563 (23.5%)	132,085 (21.2%)	30,129 (33.5%)
Initiate ezetimibe	29,367 (4.1%)	13,188 (5.0%)	910 (1.5%)	1749 (2.0%)	12,764 (4.8%)	756 (2.4%)	23,323 (3.7%)	6,044 (6.7%)
Initiate PCSK9i	10,269 (1.4%)	4,834 (1.8%)	189 (0.3%)	409 (0.5%)	4,612 (1.7%)	225 (0.7%)	8,132 (1.3%)	2,137 (2.4%)
Initiate inclisiran	264 (0.0%)	144 (0.1%)	12 (0.0%)	9 (0.0%)	97 (0.0%)	2 (0.0%)	232 (0.0%)	32 (0.0%)
Initiate bempedoic acid	418 (0.1%)	186 (0.1%)	20 (0.0%)	32 (0.0%)	173 (0.1%)	7 (0.0%)	366 (0.1%)	52 (0.1%)
Initiate bile acid sequestrants	3,222 (0.5%)	936 (0.4%)	274 (0.4%)	331 (0.4%)	1,494 (0.6%)	187 (0.6%)	2,825 (0.5%)	397 (0.4%)
Initiate any nonstatin	40,646 (5.7%)	17,950 (6.8%)	1,343 (2.2%)	2,413 (2.8%)	17,832 (6.7%)	1,108 (3.4%)	32,683 (5.3%)	7,963 (8.8%)
Initiate PCSK9, inclisiran, or bile acid sequestrants	13,590 (1.9%)	5,847 (2.2%)	469 (0.8%)	744 (0.9%)	6,123 (2.3%)	407 (1.3%)	11,049 (1.8%)	2,541 (2.8%)

Values are n (%). The table displays the number and percentage of patients who underwent specific types of therapeutic intensification within 1 year of the index date. Data are stratified by the patient's type of atherosclerotic cardiovascular disease (ASCVD) and by the occurrence of a recurrent ASCVD event within the first year of follow-up.

PCSK9i = proprotein convertase subtilisin/kexin type 9 inhibitor; other abbreviations as in Table 1.

**Figure 4:**
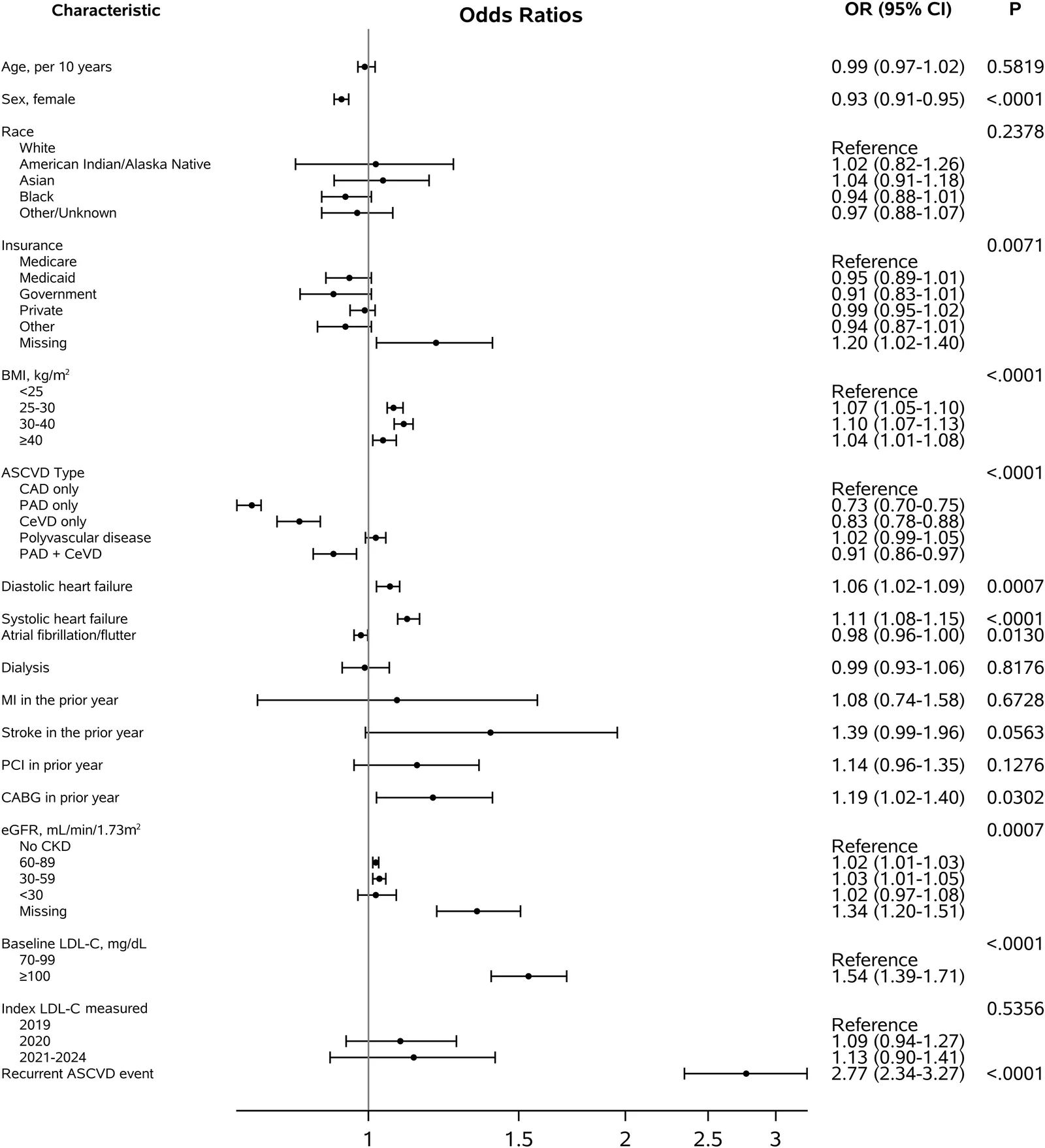
Adjusted ORs for Therapeutic Intensification Within 1 Year The forest plot displays the adjusted ORs and 95% CIs from the multivariable logistic regression model for the outcome of therapeutic intensification within 1 year of the index low-density lipoprotein cholesterol measurement. An OR >1 indicates higher odds of therapeutic intensification, while an OR < 1 indicates lower odds. The analysis included 712,057 patients. Abbreviations as in Figures 1 and 3.

In the multivariable analysis (Figure 4), a recurrent ASCVD event was the factor most strongly associated with therapeutic intensification (OR: 2.77; 95% CI: 2.34-3.27). A higher baseline LDL-C (≥100 mg/dL vs 70-99 mg/dL) was also strongly associated with increased odds of intensification (OR: 1.54; 95% CI: 1.39-1.71). Factors associated with lower odds of intensification included female sex (OR: 0.93; 95% CI: 0.91-0.95), having PAD only (OR: 0.73; 95% CI: 0.70-0.75), or CeVD only (OR: 0.83; 95% CI: 0.78-0.88).

Subgroup analyses revealed significant variation by ASCVD type (Table 2). Intensification rates were lowest among patients with PAD only (17.9%) compared to those with CAD only (27.1%) or polyvascular disease (29.6%). The initiation of any nonstatin therapy was particularly low in the PAD-only (2.2%) and CeVD-only (2.8%) groups compared to the CAD-only group (6.8%). Analysis by calendar year showed a trend toward more intensive treatment over time (Table 3). The overall intensification rate increased from 24.5% in 2019 to 29.7% in 2022. This trend was driven by an increasing use of nonstatin therapies, with the initiation rate rising from 5.5% in 2019 to 6.6% in 2023. The use of newer agents like bempedoic acid and inclisiran also emerged in 2020 and 2021, respectively.

**Table 3:** Rates of Therapeutic Intensification Within 1 Year, Stratified by Calendar Year of Index LDL-C Measurement

	2019 (n = 208,243)	2020 (n = 105,020)	2021 (n = 130,603)	2022 (n = 137,128)	2023 (n = 119,923)	2024 (n = 11,140)
Any therapeutic intensification	50,995 (24.5%)	29,117 (27.7%)	35,967 (27.5%)	40,744 (29.7%)	31,763 (26.5%)	3,112 (27.9%)
Initiate statin or dose increase	42,238 (20.3%)	24,958 (23.8%)	31,298 (24.0%)	35,162 (25.6%)	26,067 (21.7%)	2,491 (22.4%)
Initiate ezetimibe	8,040 (3.9%)	4,135 (3.9%)	4,867 (3.7%)	5,964 (4.3%)	5,705 (4.8%)	656 (5.9%)
Initiate PCSK9i	3,166 (1.5%)	1,398 (1.3%)	1,554 (1.2%)	1976 (1.4%)	1966 (1.6%)	209 (1.9%)
Initiate inclisiran	0 (0.0%)	0 (0.0%)	12 (0.0%)	103 (0.1%)	135 (0.1%)	14 (0.1%)
Initiate bempedoic acid	14 (0.0%)	59 (0.1%)	61 (0.0%)	97 (0.1%)	173 (0.1%)	14 (0.1%)
Initiate bile acid sequestrants	942 (0.5%)	460 (0.4%)	556 (0.4%)	663 (0.5%)	550 (0.5%)	51 (0.5%)
Initiate any nonstatin	11,470 (5.5%)	5,668 (5.4%)	6,606 (5.1%)	8,177 (6.0%)	7,863 (6.6%)	862 (7.7%)
Initiate PCSK9, inclisiran, or bile acid sequestrants	4,065 (2.0%)	1850 (1.8%)	2,100 (1.6%)	2,709 (2.0%)	2,597 (2.2%)	269 (2.4%)

Data are stratified by the calendar year in which the index LDL-C measurement occurred and presented as n (%). The table displays the number and percentage of patients who underwent specific types of therapeutic intensification within 1 year of the index date.

Abbreviations as in Tables 1 and 2.

### Subgroup analysis of very high-risk ASCVD

In the subgroup of patients with very high-risk ASCVD (N = 501,891), the longitudinal LDL-C trajectory followed a similar “drop-and-plateau” pattern to the overall cohort, with mean LDL-C levels stabilizing at approximately 94 mg/dL after the initial decline (Supplemental Figure 4). Similarly, multivariable logistic regression analyses within this subgroup demonstrated that factors associated with 12-month goal attainment (N = 245,571) and LLT intensification (N = 536,777) were highly consistent with the primary findings (Supplemental Figures 5 and 6). Notably, the occurrence of a recurrent ASCVD event during follow-up was the factor most strongly associated with both achieving an LDL-C <70 mg/dL (OR: 2.15; 95% CI: 1.96-2.36) and undergoing LLT intensification (OR: 2.69; 95% CI: 2.31-3.13).

### Sensitivity and bias analyses

To further contextualize the potential impact of clinically driven testing and selection bias, we performed 2 sensitivity analyses. First, within the longitudinal trajectory cohort (N = 671,353). Supplemental Table 1 compares the baseline characteristics of patients with frequent (≥1 LDL-C test/year, N = 593,523) vs infrequent (<1 test/year, N = 77,830) follow-up measurements. Compared to infrequent testers, those tested more frequently had higher rates of incident ASCVD events during follow-up (7.8% vs 5.1%) and slightly higher baseline utilization of nonstatin therapies. Additionally, within the overall baseline cohort (N = 1,033,703), Supplemental Table 2 compares the baseline characteristics of the 1-year goal attainment analysis cohort (N = 322,820) vs the excluded cohort without 1-year follow-up data (N = 710,883). Patients included in the goal attainment analysis exhibited a higher baseline prevalence of key comorbidities, such as hypertension (69.0% vs 53.4%), as well as higher baseline statin use (52.6% vs 49.5%), reflecting a cohort more actively engaged with the health care system.

## Discussion

In this large, contemporary, longitudinal study of U.S. patients with ASCVD and baseline LDL-C ≥70 mg/dL, we characterized the real-world trends in lipid management. We identified a distinct “drop-and-plateau” pattern: although mean LDL-C levels initially declined, they subsequently stalled well above guideline-recommended thresholds. Consequently, the majority of patients failed to achieve therapeutic goals; among those with available data at 3 years, over two-thirds remained above 70 mg/dL, and nearly 90% did not reach <55 mg/dL. This suboptimal control disproportionately affected women, Black patients, and those with noncoronary ASCVD. Furthermore, therapeutic intensification was infrequent, occurring in only 1 in 4 patients within the first year. Critically, a recurrent ASCVD event was the factor most strongly associated with therapeutic intensification and was strongly associated with goal attainment. These findings suggest that lipid management remains largely reactive, revealing profound therapeutic inertia among clinically “stable” outpatients.

These findings are highly consistent with contemporary cross-sectional snapshots from diverse health care settings. For instance, the European SANTORINI registry demonstrated that nearly 80% of high- and very high-risk patients fail to achieve guideline-recommended LDL-C goals, largely due to a persistent reliance on statin monotherapy and underutilization of combination therapies.^12^ Similarly, recent administrative claims analyses in the United States by Baum et al^13^ revealed that over 58% of very high-risk ASCVD patients maintained LDL-C levels ≥70 mg/dL despite LLT use. While these numerous cross-sectional studies have documented low rates of LDL-C goal attainment at fixed time points, our longitudinal modeling builds upon this foundation by providing some of the first large-scale characterization of the trends in LDL-C over time in patients followed longitudinally within a health system. While we did observe some initial improvement in LDL-C levels, these changes stall well above the 70 mg/dL threshold. While some of the initial decline may be due to initial therapeutic intensification, it may also reflect regression to the mean due to the selection of patients with an LDL-C ≥70 mg/dL at index. Nevertheless, this finding suggests that initial therapeutic efforts are often not sustained nor followed up with further titration and intensification. As a result, the current standard of care allows patients to accumulate a substantial burden of “cholesterol years,” underscoring the need for a paradigm shift that prioritizes not just initial therapy but sustained, proactive management to maintain the pace of LDL-C reduction.

Our study also highlights persistent gaps in utilization of nonstatin therapies, in particular novel nonstatin therapies. Even among individuals with ASCVD and LDL-C levels ≥70 mg/dL in 2024, only 2% were initiated on a PCSK9 inhibitor, bempedoic acid, or inclisiran within 1 year. Although ezetimibe use is increasing, <10% of patients with ASCVD and LDL-C ≥70 mg/dL were initiated on it from 2024 to 2025.

Our analysis reinforces the existence of significant care disparities that persist over the longitudinal course of lipid management. Consistent with numerous cardiovascular studies, we found that women and Black patients were significantly less likely to achieve LDL-C goals.^4^^,^^14^, ^15^, ^16^, ^17^ Furthermore, women were less likely to receive therapeutic intensification. We also found that patients with noncoronary ASCVD, such as PAD only or CeVD only, were treated less aggressively and were less likely to achieve lipid targets compared to patients with CAD only.^18^^,^^19^ What this study uniquely adds is the demonstration that these are not just point-in-time disparities; rather, they represent a sustained gap in care throughout the long-term observation period. This persistent undertreatment, particularly in the noncoronary ASCVD population, may reflect a lower perception of risk among clinicians for these conditions. These findings highlight a critical need for targeted educational interventions and system-level changes to ensure equitable and effective secondary prevention for all high-risk patient populations.

Our finding that a recurrent ASCVD event was the factor most strongly associated with therapeutic intensification underscores that real-world lipid management is often reactive and event-driven. This phenomenon is visualized in Supplemental Figure 7. While we cannot establish causality from this descriptive analysis, as unmeasured factors may influence both recurrence risk and the LDL-C trends, the figure illustrates the markedly different clinical pathways between patients who experienced a recurrent event in the first year and those who remained clinically stable. Patients with a recurrent event showed a sharp, rapid decline in mean LDL-C, demonstrating a “wake-up call” effect that triggers aggressive management. In stark contrast, clinically stable patients exhibited a much slower and less adequate decline, representing a clear pattern of therapeutic inertia. This highlights an urgent need for proactive, system-level strategies to address this inertia in clinically stable patients. Several evidence-based approaches could be implemented. For instance, integrating automated alerts or best-practice advisories into the EHR can prompt clinicians to re-evaluate lipid management for patients with persistently elevated LDL-C.^20^, ^21^, ^22^ Furthermore, multidisciplinary, team-based care has proven effective; interventions led by clinical pharmacists, who can be empowered to initiate and titrate therapies through collaborative practice agreements, have been associated with significant improvements in LDL-C control.^23^, ^24^, ^25^ By implementing such structured and proactive systems, health care can shift from a reactive to a preventive paradigm, ensuring all high-risk patients—especially those who appear clinically stable—receive the consistent and intensive treatment necessary to reduce their long-term cardiovascular risk.

### Study limitations

This study has several limitations. First, the data source consisted of deidentified EHR data from 17 U.S. academic medical centers. Patterns of testing, prescribing, and follow-up in academic systems may differ from community or rural settings, limiting generalizability. Care received outside participating systems (laboratories, prescriptions, and procedures) could be incompletely captured, potentially underestimating or overestimating LDL-C control and therapeutic intensification. Second, therapeutic intensification was defined as statin initiation or dose/intensity increase or the addition of nonstatins (ezetimibe, monoclonal antibody PCSK9 inhibitor, inclisiran, bempedoic acid, bile acid sequestrants) recorded in the EHR. Actual medication adherence, fills, dose titration details, sample use, prior-authorization denials, and out-of-network prescriptions were not available. As a result, intensification likely reflects prescribing intent rather than actual ingestion. Third, our inclusion criteria may have introduced left truncation. Time since initial ASCVD diagnosis varied, and many patients may have had long-standing exposure to LLT before their index measurement. Fourth, ASCVD diagnoses and recurrent events (myocardial infarction, stroke, percutaneous coronary intervention, and coronary artery bypass graft) were identified using EHR codes and procedure records documented within participating health systems without central adjudication. Furthermore, our finding that recurrent events drive intensification is subject to potential reverse causation; patients followed more closely by specialists are more likely to have both their medications adjusted and their events captured in the EHR. Additionally, events occurring outside participating health systems may not be captured, leading to under-ascertainment and a misclassification bias that likely attenuates the true effect estimate toward the null. This reliance on EHR and claims data from the first point of contact with the health system also meant we could not exhaustively capture remote historical ASCVD events. Consequently, our definition of “very high-risk” relied on the presence of multiple high-risk conditions rather than multiple major ASCVD events, which may slightly underestimate the true proportion of very high-risk patients in our cohort. Additionally, regarding specific high-risk conditions, the inclusion of broad or nonspecific International Classification of Diseases codes to define PAD (eg, I70.0, I70.1, I73.9) may have captured distinct clinical syndromes or nonatherosclerotic conditions, potentially misclassifying some patients and inflating the prevalence of this specific subgroup. Fifth, because follow-up testing was clinically driven, the observation process is likely informative: patients tested more frequently were likely those receiving more intensive management or closer surveillance. Our analysis suggests our population-level trajectory may represent a “best-case scenario” of the most engaged patients, meaning true therapeutic inertia is likely even more profound across the broader real-world population. Sixth, our 12-month goal attainment cohort represented roughly one-third of the baseline population. Those without follow-up labs were excluded, likely representing patients less engaged with the health care system. Consequently, our reported 23% goal attainment rate likely overestimates true systemic control across the broader population. Seventh, for Sankey diagrams, we used nonmutually exclusive windows (1-13, 13-25, 25-37 months) and selected the last LDL-C value within each window. This pragmatic approach improved comparability but may have been influenced by clinic visit timing, potentially over-representing worse or better values depending on care patterns. Finally, we did not account for death or disenrollment from participating health systems as competing risks. Patients with poor LDL-C control may have been more likely to die or be lost to follow-up, biasing later LDL-C distributions toward survivors with better control. Conversely, clinically stable patients with well-controlled LDL-C may be less likely to return to tertiary academic centers for follow-up, potentially biasing the observed trends toward higher LDL-C levels.

## Conclusions

In this large, real-world cohort of patients with ASCVD and elevated baseline LDL-C, longitudinal lipid trajectories reveal an initial improvement followed by a persistent plateau well above guideline-recommended targets. Therapeutic inertia emerges as a central barrier to optimal care, particularly among clinically stable patients without recurrent events. Notably, disparities persist, with women and patients with noncoronary ASCVD experiencing lower rates of goal attainment and treatment intensification. These findings underscore the need to shift from reactive, event-driven treatment toward proactive, goal-directed strategies that prevent prolonged cumulative exposure to uncontrolled LDL-C.

## Funding support and author disclosures

This study was funded by Amgen. Dr Freeman is supported by research funding from Amgen, Novartis, NIH, and the American Diabetes Association. Dr Shah reports consulting for Novartis, Amgen, Amarin, and Merck, and research support from the NIH, Amgen, Novartis, and Janssen. Dr Bittner reports research support to my institution as the site PI ORION IV trial (Novartis); site PI EVOLVE MI trial (Amgen); also serves on DSMB for Verve Therapeutics, Eli Lilly, and the NIH, is an editor for the American College of Cardiology, ACCSAP; and is a Senior Guest Editor for Circulation at American Heart Association. Dr Wilcox has received support from Novo Nordisk (clinical advisory board). Drs Wójcik, Jin, and Jones are employees of and stockholders in Amgen Inc. Dr Peterson reports research grant support from Amgen and Esperion, personal support from Janssen (clinical study advisory board), and is a member of the FDA cardiorenal advisory board. Dr Navar reports consulting for AstraZeneca, Bayer, BMS, Boehringer Ingelheim, Eli Lilly, Esperion, Janssen, Merck, New Amsterdam, Novo Nordisk, Novartis, Silence Therapeutics, and Pfizer, and research support to my institution from Janssen, Esperion, and Amgen. All other authors have reported that they have no relationships relevant to the contents of this paper to disclose.
