# Supplementary material for: Event-Driven Intensification and Persistent Therapeutic Inertia in LDL-C Management Among 1,033,703 Patients With ASCVD
Source: JACC Adv. 2026 Aug 22;5(9):103160. doi: 10.1016/j.jacadv.2026.103160 (PMC13524565; doi:10.1016/j.jacadv.2026.103160)
Supplement: Supplementary file 1 [file mmc1.docx]

**Supplemental Materials**

**Supplemental Figure 1. Mean Longitudinal LDL-C Trajectory in Patients with Historically Sustained LDL-C Elevations**

The solid line represents the estimated mean LDL-C trajectory from a generalized estimating equation model using restricted cubic splines, restricted to a sub-cohort of patients with at least one prior documented LDL-C ≥70 mg/dL within 12 months before the index measurement; the shaded band represents the 95% confidence interval. The black dashed horizontal line indicates the guideline-recommended goal of <70 mg/dL. The analysis included 208,836 patients.

**Supplemental Figure 2. Mean Longitudinal LDL-C Trajectory, Stratified by Sex**

The lines represent the estimated mean LDL-C trajectories from a generalized estimating equation model using restricted cubic splines, stratified by patient sex (the solid red line represents females and the solid blue line represents males); the shaded bands represent the 95% confidence intervals. The black dashed horizontal line indicates the guideline-recommended goal of <70 mg/dL. The analysis included 671,353 patients.

**Abbreviations:** LDL-C, low-density lipoprotein cholesterol.

**Supplemental Figure 3. Mean Longitudinal LDL-C Trajectory, Stratified by Race**

The lines represent the estimated mean LDL-C trajectories from a generalized estimating equation model using restricted cubic splines, stratified by patient race (White, Black, Asian, American Indian/Alaska Native, and Other/Missing) using solid lines of distinct colors; the shaded bands represent the 95% confidence intervals. The black dashed horizontal line indicates the guideline-recommended goal of <70 mg/dL. The analysis included 671,353 patients.

**Abbreviations:** LDL-C, low-density lipoprotein cholesterol.

**Supplemental Figure 4. Mean Longitudinal LDL-C Trajectory in the Very High-Risk ASCVD Subgroup**

The line represents the estimated mean LDL-C trajectory from a generalized estimating equation model using restricted cubic splines for the prespecified subgroup of patients with very high-risk atherosclerotic cardiovascular disease (ASCVD); the shaded band represents the 95% confidence interval. The black dashed horizontal line indicates the guideline-recommended goal of <70 mg/dL. Very high-risk ASCVD was defined as having an established ASCVD diagnosis plus two or more high-risk conditions at baseline. The analysis included 501,891 patients.

**Abbreviations:** ASCVD = atherosclerotic cardiovascular disease; LDL-C = low-density lipoprotein cholesterol.

**Supplemental Figure 5. Factors Associated with Achieving LDL-C <70 mg/dL at 12 Months in the Very High-Risk ASCVD Subgroup**

The forest plot displays the adjusted odds ratios (ORs) and 95% confidence intervals (CIs) from the multivariable logistic regression model for the outcome of achieving an LDL-C level <70 mg/dL at 12 months (6-18 months post-baseline) among the prespecified subgroup of patients with very high-risk ASCVD. An OR > 1 indicates higher odds of achieving the goal, while an OR < 1 indicates lower odds. The analysis included 245,571 patients.

**Abbreviations:** ASCVD, atherosclerotic cardiovascular disease; BMI, body mass index; CABG, coronary artery bypass grafting; CAD, coronary artery disease; CeVD, cerebrovascular disease; CI, confidence interval; eGFR, estimated glomerular filtration rate; LDL-C, low-density lipoprotein cholesterol; MI, myocardial infarction; OR, odds ratio; PAD, peripheral artery disease; PCI, percutaneous coronary intervention.

**Supplemental Figure 6. Factors Associated with Lipid-Lowering Therapy Intensification Within 1 Year in the Very High-Risk ASCVD Subgroup**

The forest plot displays the adjusted odds ratios (ORs) and 95% confidence intervals (CIs) from the multivariable logistic regression model for the outcome of any lipid-lowering therapy (LLT) intensification within 1 year post-baseline among the prespecified subgroup of patients with very high-risk ASCVD. An OR > 1 indicates higher odds of undergoing intensification, while an OR < 1 indicates lower odds. The very high-risk subgroup for this intensification analysis (N=536,777) was derived from the overall baseline cohort, whereas the subgroup for the longitudinal trajectory analysis (N=501,891) was restricted to those with available follow-up LDL-C measurements.

**Abbreviations:** ASCVD, atherosclerotic cardiovascular disease; BMI, body mass index; CABG, coronary artery bypass grafting; CAD, coronary artery disease; CeVD, cerebrovascular disease; CI, confidence interval; eGFR, estimated glomerular filtration rate; LDL-C, low-density lipoprotein cholesterol; LLT, lipid-lowering therapy; MI, myocardial infarction; OR, odds ratio; PAD, peripheral artery disease; PCI, percutaneous coronary intervention.

**Supplemental Figure 7. Mean Longitudinal LDL-C Trajectory, Stratified by Recurrent ASCVD Event in the First Year**

The lines represent the estimated mean LDL-C trajectories from a generalized estimating equation model using restricted cubic splines, stratified by the occurrence of a recurrent ASCVD event within the first year of follow-up (the solid red line represents patients who experienced a recurrent event, and the solid blue line represents those who did not); the shaded bands represent the 95% confidence intervals. The black dashed horizontal line indicates the guideline-recommended goal of <70 mg/dL. This descriptive analysis visualizes the different trajectories of two clinically distinct groups.

**Abbreviations:** ASCVD, atherosclerotic cardiovascular disease; LDL-C, low-density lipoprotein cholesterol.

**Supplemental Table 1. Baseline Characteristics of the Longitudinal Trajectory Cohort Stratified by LDL-C Testing Frequency**

| **Characteristic** | **Infrequent Testing  (<1 LDL-C test/year)** | **Frequent Testing  (≥1 LDL-C test/year)** | **P** |
| --- | --- | --- | --- |
| N | 77,830 | 593,523 |  |
| **Demographics** |  |  |  |
| Age at index date, yrs |  |  | <.0001 |
| Median (Q1, Q3) | 67.3 (58.8, 75.3) | 68.2 (60.2, 75.5) |  |
| Mean (SD) | 66.6 (12.2) | 67.5 (11.7) |  |
| Range | 18.9, 106.4 | 19.7, 124.5 |  |
| Age ≥65 | 44700 (57.4%) | 365044 (61.5%) | <.001 |
| Female | 36541 (47.0%) | 291134 (49.1%) | <.001 |
| Missing | 73 (0.1%) | 793 (0.1%) |  |
| Race |  |  | <.001 |
| American Indian/Alaska Native | 220 (0.3%) | 1478 (0.2%) |  |
| Asian | 932 (1.2%) | 9380 (1.6%) |  |
| Black | 13061 (16.8%) | 90221 (15.2%) |  |
| White | 60823 (78.1%) | 469339 (79.1%) |  |
| Other | 227 (0.3%) | 1944 (0.3%) |  |
| Unknown/Missing | 2567 (3.3%) | 21161 (3.6%) |  |
| Hispanic Ethnicity | 2822 (3.8%) | 25290 (4.6%) | <.001 |
| Missing | 3743 (4.8%) | 46887 (7.9%) |  |
| Insurance |  |  | <.001 |
| Medicare | 32816 (42.9%) | 286227 (49.0%) |  |
| Medicaid | 3419 (4.5%) | 20843 (3.6%) |  |
| Government | 907 (1.2%) | 7510 (1.3%) |  |
| Private | 18911 (24.7%) | 144425 (24.7%) |  |
| Other | 13984 (18.3%) | 63554 (10.9%) |  |
| None/missing | 6491 (8.5%) | 61923 (10.6%) |  |
| Missing | 1302 (1.7%) | 9041 (1.5%) |  |
|  |  |  |  |
| **Smoking Status** |  |  |  |
| Smoking status |  |  |  |
| Current | 10356 (13.3%) | 69997 (11.8%) |  |
| Former | 18633 (23.9%) | 145346 (24.5%) |  |
| Never | 29300 (37.6%) | 240854 (40.6%) |  |
| Unknown/Missing | 19541 (25.1%) | 137326 (23.1%) |  |
|  |  |  |  |
| **Type of ASCVD** |  |  |  |
| Type of ASCVD |  |  | <.001 |
| CAD only | 41557 (53.4%) | 298134 (50.2%) |  |
| PAD only | 8973 (11.5%) | 86045 (14.5%) |  |
| CeVD only | 13715 (17.6%) | 108082 (18.2%) |  |
| Polyvascular disease | 11969 (15.4%) | 88930 (15.0%) |  |
| PAD + CeVD | 1616 (2.1%) | 12332 (2.1%) |  |
|  |  |  |  |
| **Comorbidities** |  |  |  |
| Dialysis | 1314 (1.7%) | 9711 (1.6%) | 0.282 |
| Atrial fibrillation or flutter | 13585 (17.5%) | 91532 (15.4%) | <.001 |
| Familial hypercholesterolemia | 403 (0.5%) | 4400 (0.7%) | <.001 |
| Liver disease | 5893 (7.6%) | 48267 (8.1%) | <.001 |
| Systolic HF | 8068 (10.4%) | 52781 (8.9%) | <.001 |
| Diastolic HF | 6855 (8.8%) | 44652 (7.5%) | <.001 |
| Hypertension | 53868 (69.2%) | 395182 (66.6%) | <.001 |
| HIV | 419 (0.5%) | 3475 (0.6%) | 0.103 |
| Psoriasis | 1344 (1.7%) | 10129 (1.7%) | 0.682 |
| Rheumatoid arthritis | 2023 (2.6%) | 14104 (2.4%) | <.001 |
|  |  |  |  |
| **Diabetes-Specific Complications** |  |  |  |
| Retinopathy | 2068 (2.7%) | 18454 (3.1%) | <.001 |
| Neuropathy | 5755 (7.4%) | 51857 (8.7%) | <.001 |
|  |  |  |  |
| **Events in the Past 1 Year** |  |  |  |
| MI hospitalization | 4120 (5.3%) | 31850 (5.4%) | 0.397 |
| Stroke hospitalization | 3716 (4.8%) | 28223 (4.8%) | 0.812 |
| CABG | 555 (0.7%) | 4538 (0.8%) | 0.120 |
| PCI | 2148 (2.8%) | 16486 (2.8%) | 0.776 |
|  |  |  |  |
| **Vitals and Labs** |  |  |  |
| BMI, kg/m^2^ |  |  | <.0001 |
| Median (Q1, Q3) | 29.1 (25.6, 33.8) | 29.4 (25.8, 34.0) |  |
| Mean (SD) | 30.2 (6.9) | 30.4 (6.9) |  |
| Range | 10.6, 84.2 | 10.0, 84.5 |  |
| Missing | 3426 (4.4%) | 20528 (3.5%) |  |
| LDL-C, mg/dL |  |  | <.0001 |
| Median (Q1, Q3) | 97.0 (82.0, 120.0) | 99.0 (83.0, 124.0) |  |
| Mean (SD) | 104.8 (30.3) | 107.5 (32.6) |  |
| Range | 70.0, 482.0 | 70.0, 498.0 |  |
| HDL-C, mg/dL |  |  | <.0001 |
| Median (Q1, Q3) | 48.0 (40.0, 61.0) | 48.0 (40.0, 60.0) |  |
| Mean (SD) | 53.2 (21.6) | 52.2 (19.6) |  |
| Range | 5.0, 200.0 | 2.0, 200.0 |  |
| Missing | 3401 (4.4%) | 43537 (7.3%) |  |
| Non HDL-C, mg/dL |  |  | <.0001 |
| Median (Q1, Q3) | 123.0 (104.0, 149.0) | 126.0 (105.0, 155.0) |  |
| Mean (SD) | 130.5 (36.4) | 134.2 (40.0) |  |
| Range | 3.0, 397.0 | 2.0, 400.0 |  |
| Missing | 38600 (49.6%) | 306881 (51.7%) |  |
| Triglycerides, mg/dL |  |  | <.0001 |
| Median (Q1, Q3) | 116.0 (84.0, 164.0) | 119.0 (86.0, 169.0) |  |
| Mean (SD) | 137.1 (91.7) | 142.2 (100.6) |  |
| Range | 11.0, 4409.0 | 11.0, 5000.0 |  |
| Missing | 2436 (3.1%) | 28929 (4.9%) |  |
| Total cholesterol, mg/dL |  |  | <.0001 |
| Median (Q1, Q3) | 175.0 (153.0, 203.0) | 177.0 (154.0, 208.0) |  |
| Mean (SD) | 180.3 (41.6) | 183.3 (46.1) |  |
| Range | 1.8, 831.0 | 1.7, 975.0 |  |
| Missing | 1222 (1.6%) | 14046 (2.4%) |  |
| HbA1c, % |  |  | <.0001 |
| Median (Q1, Q3) | 5.9 (5.5, 6.8) | 6.0 (5.6, 7.0) |  |
| Mean (SD) | 6.4 (1.6) | 6.5 (1.7) |  |
| Range | 3.5, 19.8 | 3.2, 20.0 |  |
| Missing | 37194 (47.8%) | 233301 (39.3%) |  |
| HbA1c <7 | 31558 (77.7%) | 269534 (74.8%) | <.001 |
| eGFR, mL/min/1.73m^2^ |  |  | <.0001 |
| Median (Q1, Q3) | 76.0 (61.0, 91.0) | 76.0 (60.0, 90.0) |  |
| Mean (SD) | 74.5 (21.6) | 73.7 (21.6) |  |
| Range | 4.0, 238.0 | 4.0, 238.0 |  |
| Missing | 3132 (4.0%) | 17064 (2.9%) |  |
| Chronic Kidney Disease |  |  | <.001 |
| Grade 1: eGFR ≥90 | 20378 (27.2%) | 150649 (26.1%) |  |
| Grade 2: eGFR 60-90 | 36501 (48.8%) | 283245 (49.1%) |  |
| Grade 3: eGFR 30-60 | 14831 (19.8%) | 118167 (20.5%) |  |
| Grade 4: eGFR 15-30 | 1053 (1.4%) | 9602 (1.7%) |  |
| Grade 5: On dialysis or eGFR <15 | 2079 (2.8%) | 15794 (2.7%) |  |
|  |  |  |  |
| **Baseline Lipid Lowering Therapy** |  |  |  |
| Statin | 40635 (52.2%) | 309196 (52.1%) | 0.546 |
| Statin intensity |  |  | <.001 |
| Low | 39767 (51.1%) | 304786 (51.4%) |  |
| Moderate | 17052 (21.9%) | 136774 (23.0%) |  |
| High | 21011 (27.0%) | 151963 (25.6%) |  |
| Ezetimibe | 2233 (2.9%) | 23102 (3.9%) | <.001 |
| PCSK9i | 477 (0.6%) | 5303 (0.9%) | <.001 |
| **New ASCVD Events** |  |  |  |
| Any event | 3954 (5.1%) | 46575 (7.8%) | <.001 |
| MI | 1198 (1.5%) | 14765 (2.5%) | <.001 |
| Stroke | 1235 (1.6%) | 17435 (2.9%) | <.001 |
| PCI | 1206 (1.5%) | 12376 (2.1%) | <.001 |
| CABG | 917 (1.2%) | 10308 (1.7%) | <.001 |

This table compares the baseline characteristics of patients within the longitudinal trajectory cohort (N=671,353) who had frequent (≥1 LDL-C test/year, N=593,523) versus infrequent (<1 LDL-C test/year, N=77,830) follow-up measurements to assess potential informative observation bias. Continuous variables are presented as medians (Q1, Q3) and means (SD). Categorical variables are presented as counts (percentages).

**Abbreviations:** ASCVD = atherosclerotic cardiovascular disease; CAD = coronary artery disease; CeVD = cerebrovascular disease; eGFR = estimated glomerular filtration rate; HbA1c = glycated hemoglobin; IQR = interquartile range; LDL-C = low-density lipoprotein cholesterol; PAD = peripheral artery disease; Q1 = first quartile; Q3 = third quartile; SD = standard deviation.

**Supplemental Table 2. Baseline Characteristics of the Overall Cohort Stratified by 12-Month LDL-C Data Availability**

| **Characteristic** | **Excluded  (No LDL-C  at 12 Months)** | **Included  (LDL-C Available  at 12 Months)** | **P** |
| --- | --- | --- | --- |
| N | 710,883 | 322,820 |  |
| **Demographics** |  |  |  |
| Age at index date, yrs |  |  | <.0001 |
| Median (Q1, Q3) | 67.5 (58.6, 75.7) | 68.8 (61.1, 75.8) |  |
| Mean (SD) | 66.7 (12.9) | 68.0 (11.3) |  |
| Range | 18.5, 121.8 | 19.7, 124.5 |  |
| Age ≥65 | 411613 (57.9%) | 206823 (64.1%) | <.001 |
| Female | 349596 (49.2%) | 158522 (49.2%) | 0.711 |
| Missing | 585 (0.1%) | 482 (0.1%) |  |
| Race |  |  | <.001 |
| American Indian/Alaska Native | 2135 (0.3%) | 728 (0.2%) |  |
| Asian | 11419 (1.6%) | 4966 (1.5%) |  |
| Black | 117585 (16.5%) | 46263 (14.3%) |  |
| White | 544358 (76.6%) | 259401 (80.4%) |  |
| Other | 2888 (0.4%) | 936 (0.3%) |  |
| Unknown/Missing | 32497 (4.6%) | 10526 (3.3%) |  |
| Hispanic Ethnicity | 34398 (5.3%) | 12903 (4.3%) | <.001 |
| Missing | 66271 (9.3%) | 24400 (7.6%) |  |
| Insurance |  |  | <.001 |
| Medicare | 316023 (45.1%) | 161396 (50.7%) |  |
| Medicaid | 33145 (4.7%) | 9386 (2.9%) |  |
| Government | 12315 (1.8%) | 3650 (1.1%) |  |
| Private | 165039 (23.5%) | 76579 (24.1%) |  |
| Other | 86205 (12.3%) | 35747 (11.2%) |  |
| None/missing | 88438 (12.6%) | 31439 (9.9%) |  |
| Missing | 9718 (1.4%) | 4623 (1.4%) |  |
|  |  |  |  |
| **Smoking Status** |  |  |  |
| Smoking status |  |  | <.001 |
| Current | 93929 (13.2%) | 34898 (10.8%) |  |
| Former | 151987 (21.4%) | 82329 (25.5%) |  |
| Never | 265313 (37.3%) | 135325 (41.9%) |  |
| Unknown/Missing | 199654 (28.1%) | 70268 (21.8%) |  |
|  |  |  |  |
| **Type of ASCVD** |  |  |  |
| Type of ASCVD |  |  | <.001 |
| CAD only | 343958 (48.4%) | 162509 (50.3%) |  |
| PAD only | 88155 (12.4%) | 51954 (16.1%) |  |
| CeVD only | 152619 (21.5%) | 55971 (17.3%) |  |
| Polyvascular disease | 110798 (15.6%) | 45824 (14.2%) |  |
| PAD + CeVD | 15353 (2.2%) | 6562 (2.0%) |  |
|  |  |  |  |
| **Comorbidities** |  |  |  |
| Dialysis | 10745 (1.5%) | 4824 (1.5%) | 0.507 |
| Atrial fibrillation or flutter | 91895 (12.9%) | 50811 (15.7%) | <.001 |
| Familial hypercholesterolemia | 2905 (0.4%) | 2669 (0.8%) | <.001 |
| Liver disease | 43145 (6.1%) | 27009 (8.4%) | <.001 |
| Systolic HF | 54731 (7.7%) | 26814 (8.3%) | <.001 |
| Diastolic HF | 43779 (6.2%) | 23617 (7.3%) | <.001 |
| Hypertension | 379884 (53.4%) | 222683 (69.0%) | <.001 |
| HIV | 3184 (0.4%) | 1911 (0.6%) | <.001 |
| Psoriasis | 8258 (1.2%) | 5960 (1.8%) | <.001 |
| Rheumatoid arthritis | 13556 (1.9%) | 7800 (2.4%) | <.001 |
|  |  |  |  |
| **Diabetes-Specific Complications** |  |  |  |
| Retinopathy | 14587 (2.1%) | 10237 (3.2%) | <.001 |
| Neuropathy | 41457 (5.8%) | 29094 (9.0%) |  |
|  |  |  |  |
| **Events in the Past 1 Year** |  |  |  |
| MI hospitalization | 49115 (6.9%) | 12458 (3.9%) | <.001 |
| Stroke hospitalization | 60409 (8.5%) | 9994 (3.1%) | <.001 |
| CABG | 6060 (0.9%) | 2128 (0.7%) | <.001 |
| PCI | 21839 (3.1%) | 7028 (2.2%) | <.001 |
|  |  |  |  |
| **Vitals and Labs** |  |  |  |
| BMI, kg/m^2^ |  |  | <.001 |
| Median (Q1, Q3) | 29.0 (25.2, 33.8) | 29.3 (25.8, 34.0) |  |
| Mean (SD) | 30.1 (7.1) | 30.4 (6.7) |  |
| Range | 10.0, 84.7 | 10.7, 84.5 |  |
| Missing | 34516 (4.9%) | 9759 (3.0%) |  |
| LDL-C, mg/dL |  |  | <.001 |
| Median (Q1, Q3) | 100.0 (83.0, 125.0) | 98.0 (82.0, 122.0) |  |
| Mean (SD) | 108.0 (32.5) | 105.9 (31.6) |  |
| Range | 70.0, 498.0 | 70.0, 498.0 |  |
| HDL-C, mg/dL |  |  | <.001 |
| Median (Q1, Q3) | 48.0 (39.0, 60.0) | 49.0 (40.0, 60.0) |  |
| Mean (SD) | 52.0 (20.4) | 52.6 (19.3) |  |
| Range | 2.0, 200.0 | 3.0, 200.0 |  |
| Missing | 70689 (9.9%) | 22244 (6.9%) |  |
| Non HDL-C, mg/dL |  |  | <.001 |
| Median (Q1, Q3) | 126.0 (105.0, 154.0) | 124.0 (104.0, 152.0) |  |
| Mean (SD) | 133.5 (39.5) | 132.0 (38.7) |  |
| Range | 2.0, 400.0 | 2.0, 400.0 |  |
| Missing | 344688 (48.5%) | 164167 (50.9%) |  |
| Triglycerides, mg/dL |  |  | <.001 |
| Median (Q1, Q3) | 116.0 (83.0, 165.0) | 118.0 (85.0, 167.0) |  |
| Mean (SD) | 138.6 (97.2) | 140.1 (96.7) |  |
| Range | 11.0, 4939.0 | 12.0, 5000.0 |  |
| Missing | 51354 (7.2%) | 15084 (4.7%) |  |
| Total cholesterol, mg/dL |  |  | <.001 |
| Median (Q1, Q3) | 177.0 (154.0, 207.0) | 176.0 (154.0, 206.0) |  |
| Mean (SD) | 182.5 (45.3) | 181.9 (45.2) |  |
| Range | 1.7, 975.0 | 1.7, 968.0 |  |
| Missing | 29616 (4.2%) | 6709 (2.1%) |  |
| HbA1c, % |  |  | <.001 |
| Median (Q1, Q3) | 5.9 (5.5, 6.7) | 6.0 (5.6, 6.9) |  |
| Mean (SD) | 6.4 (1.7) | 6.5 (1.6) |  |
| Range | 3.2, 20.0 | 3.2, 19.9 |  |
| Missing | 262864 (37.0%) | 133589 (41.4%) |  |
| HbA1c <7 | 348993 (77.9%) | 142145 (75.1%) |  |
| eGFR, mL/min/1.73m^2^ |  |  | <.001 |
| Median (Q1, Q3) | 76.0 (60.0, 91.0) | 76.0 (60.0, 90.0) |  |
| Mean (SD) | 74.0 (22.9) | 73.8 (20.8) |  |
| Range | 4.0, 238.0 | 4.0, 238.0 |  |
| Missing | 25497 (3.6%) | 8404 (2.6%) |  |
| Chronic Kidney Disease |  |  | <.001 |
| Grade 1: eGFR ≥90 | 193057 (28.1%) | 79860 (25.4%) |  |
| Grade 2: eGFR 60-90 | 319667 (46.5%) | 158834 (50.4%) |  |
| Grade 3: eGFR 30-60 | 139861 (20.4%) | 63922 (20.3%) |  |
| Grade 4: eGFR 15-30 | 14712 (2.1%) | 4441 (1.4%) |  |
| Grade 5: On dialysis or eGFR <15 | 19522 (2.8%) | 7801 (2.5%) |  |
|  |  |  |  |
| **Baseline Lipid Lowering Therapy** |  |  |  |
| Statin | 351657 (49.5%) | 169690 (52.6%) | <.001 |
| Statin intensity |  |  | <.001 |
| Low | 377465 (53.1%) | 165544 (51.3%) |  |
| Moderate | 141300 (19.9%) | 78957 (24.5%) |  |
| High | 192118 (27.0%) | 78319 (24.3%) |  |
| Ezetimibe | 23169 (3.3%) | 12762 (4.0%) | <.001 |
| PCSK9i | 5288 (0.7%) | 2823 (0.9%) | <.001 |
|  |  |  |  |
| **New ASCVD Events** |  |  |  |
| Any event | 56472 (7.9%) | 19307 (6.0%) | <.001 |
| MI | 15602 (2.2%) | 6449 (2.0%) | <.001 |
| Stroke | 22511 (3.2%) | 6827 (2.1%) | <.001 |
| PCI | 13062 (1.8%) | 5506 (1.7%) | <.001 |
| CABG | 13317 (1.9%) | 4031 (1.2%) | <.001 |

This table compares the baseline characteristics of patients within the overall baseline cohort (N=1,033,703) who were included in the 12-month goal attainment analysis (LDL-C available at 12 months, N=322,820) versus those who were excluded (no LDL-C available at 12 months, N=710,883) to evaluate potential selection bias. Continuous variables are presented as medians (Q1, Q3) and means (SD). Categorical variables are presented as counts (percentages).

**Abbreviations:** ASCVD = atherosclerotic cardiovascular disease; CAD = coronary artery disease; CeVD = cerebrovascular disease; eGFR = estimated glomerular filtration rate; HbA1c = glycated hemoglobin; IQR = interquartile range; LDL-C = low-density lipoprotein cholesterol; PAD = peripheral artery disease; Q1 = first quartile; Q3 = third quartile; SD = standard deviation.

**Supplemental Appendix. ICD-10 Diagnosis and Procedure Codes Used to Define Clinical Atherosclerotic Cardiovascular Disease**

| ICD10 Code | Code Description |
| --- | --- |
| I20.0 | UNSTABLE ANGINA |
| I20.8 | OTHER FORMS OF ANGINA PECTORIS |
| I20.9 | ANGINA PECTORIS, UNSPECIFIED |
| I21 | ACUTE MYOCARDIAL INFARCTION |
| I21.0 | ST ELEVATION (STEMI) MYOCARDIAL INFARCTION OF ANTERIOR WALL |
| I21.01 | ST ELEVATION (STEMI) MYOCARDIAL INFARCTION INVOLVING LEFT MAIN CORONARY ARTERY |
| I21.02 | ST ELEVATION (STEMI) MYOCARDIAL INFARCTION INVOLVING LEFT ANTERIOR DESCENDING CORONARY ARTERY |
| I21.09 | ST ELEVATION (STEMI) MYOCARDIAL INFARCTION INVOLVING OTHER CORONARY ARTERY OF ANTERIOR WALL |
| I21.1 | ST ELEVATION (STEMI) MYOCARDIAL INFARCTION OF INFERIOR WALL |
| I21.11 | ST ELEVATION (STEMI) MYOCARDIAL INFARCTION INVOLVING RIGHT CORONARY ARTERY |
| I21.19 | ST ELEVATION (STEMI) MYOCARDIAL INFARCTION INVOLVING OTHER CORONARY ARTERY OF INFERIOR WALL |
| I21.2 | ST ELEVATION (STEMI) MYOCARDIAL INFARCTION OF OTHER SITES |
| I21.21 | ST ELEVATION (STEMI) MYOCARDIAL INFARCTION INVOLVING LEFT CIRCUMFLEX CORONARY ARTERY |
| I21.29 | ST ELEVATION (STEMI) MYOCARDIAL INFARCTION INVOLVING OTHER SITES |
| I21.3 | ST ELEVATION (STEMI) MYOCARDIAL INFARCTION OF UNSPECIFIED SITE |
| I21.4 | NON-ST ELEVATION (NSTEMI) MYOCARDIAL INFARCTION |
| I21.9 | ACUTE MYOCARDIAL INFARCTION, UNSPECIFIED |
| I21.A | OTHER TYPE OF MYOCARDIAL INFARCTION |
| I22 | SUBSEQUENT ST ELEVATION (STEMI) AND NON-ST ELEVATION (NSTEMI) MYOCARDIAL INFARCTION |
| I22.0 | SUBSEQUENT ST ELEVATION (STEMI) MYOCARDIAL INFARCTION OF ANTERIOR WALL |
| I22.1 | SUBSEQUENT ST ELEVATION (STEMI) MYOCARDIAL INFARCTION OF INFERIOR WALL |
| I22.2 | SUBSEQUENT NON-ST ELEVATION (NSTEMI) MYOCARDIAL INFARCTION |
| I22.8 | SUBSEQUENT ST ELEVATION (STEMI) MYOCARDIAL INFARCTION OF OTHER SITES |
| I22.9 | SUBSEQUENT ST ELEVATION (STEMI) MYOCARDIAL INFARCTION OF UNSPECIFIED SITE |
| I24 | OTHER ACUTE ISCHEMIC HEART DISEASES |
| I24.0 | ACUTE CORONARY THROMBOSIS NOT RESULTING IN MYOCARDIAL INFARCTION |
| I24.1 | DRESSLER'S SYNDROME |
| I24.8 | OTHER FORMS OF ACUTE ISCHEMIC HEART DISEASE |
| I24.9 | ACUTE ISCHEMIC HEART DISEASE, UNSPECIFIED |
| I25 | CHRONIC ISCHEMIC HEART DISEASE |
| I25.1 | ATHEROSCLEROTIC HEART DISEASE OF NATIVE CORONARY ARTERY |
| I25.10 | ATHEROSCLEROTIC HEART DISEASE OF NATIVE CORONARY ARTERY WITHOUT ANGINA PECTORIS |
| I25.11 | ATHEROSCLEROTIC HEART DISEASE OF NATIVE CORONARY ARTERY WITH ANGINA PECTORIS |
| I25.110 | ATHEROSCLEROTIC HEART DISEASE OF NATIVE CORONARY ARTERY WITH UNSTABLE ANGINA PECTORIS |
| I25.111 | ATHEROSCLEROTIC HEART DISEASE OF NATIVE CORONARY ARTERY WITH ANGINA PECTORIS WITH DOCUMENTED SPASM |
| I25.118 | ATHEROSCLEROTIC HEART DISEASE OF NATIVE CORONARY ARTERY WITH OTHER FORMS OF ANGINA PECTORIS |
| I25.119 | ATHEROSCLEROTIC HEART DISEASE OF NATIVE CORONARY ARTERY WITH UNSPECIFIED ANGINA PECTORIS |
| I25.2 | OLD MYOCARDIAL INFARCTION |
| I25.3 | ANEURYSM OF HEART |
| I25.5 | ISCHEMIC CARDIOMYOPATHY |
| I25.6 | SILENT MYOCARDIAL ISCHEMIA |
| I25.7 | ATHEROSCLEROSIS OF CORONARY ARTERY BYPASS GRAFT(S) AND CORONARY ARTERY OF TRANSPLANTED HEART WITH ANGINA PECTORIS |
| I25.70 | ATHEROSCLEROSIS OF CORONARY ARTERY BYPASS GRAFT(S), UNSPECIFIED, WITH ANGINA PECTORIS |
| I25.700 | ATHEROSCLEROSIS OF CORONARY ARTERY BYPASS GRAFT(S), UNSPECIFIED, WITH UNSTABLE ANGINA PECTORIS |
| I25.701 | ATHEROSCLEROSIS OF CORONARY ARTERY BYPASS GRAFT(S), UNSPECIFIED, WITH ANGINA PECTORIS WITH DOCUMENTED SPASM |
| I25.708 | ATHEROSCLEROSIS OF CORONARY ARTERY BYPASS GRAFT(S), UNSPECIFIED, WITH OTHER FORMS OF ANGINA PECTORIS |
| I25.709 | ATHEROSCLEROSIS OF CORONARY ARTERY BYPASS GRAFT(S), UNSPECIFIED, WITH UNSPECIFIED ANGINA PECTORIS |
| I25.71 | ATHEROSCLEROSIS OF AUTOLOGOUS VEIN CORONARY ARTERY BYPASS GRAFT(S) WITH ANGINA PECTORIS |
| I25.710 | ATHEROSCLEROSIS OF AUTOLOGOUS VEIN CORONARY ARTERY BYPASS GRAFT(S) WITH UNSTABLE ANGINA PECTORIS |
| I25.711 | ATHEROSCLEROSIS OF AUTOLOGOUS VEIN CORONARY ARTERY BYPASS GRAFT(S) WITH ANGINA PECTORIS WITH DOCUMENTED SPASM |
| I25.718 | ATHEROSCLEROSIS OF AUTOLOGOUS VEIN CORONARY ARTERY BYPASS GRAFT(S) WITH OTHER FORMS OF ANGINA PECTORIS |
| I25.719 | ATHEROSCLEROSIS OF AUTOLOGOUS VEIN CORONARY ARTERY BYPASS GRAFT(S) WITH UNSPECIFIED ANGINA PECTORIS |
| I25.72 | ATHEROSCLEROSIS OF AUTOLOGOUS ARTERY CORONARY ARTERY BYPASS GRAFT(S) WITH ANGINA PECTORIS |
| I25.720 | ATHEROSCLEROSIS OF AUTOLOGOUS ARTERY CORONARY ARTERY BYPASS GRAFT(S) WITH UNSTABLE ANGINA PECTORIS |
| I25.721 | ATHEROSCLEROSIS OF AUTOLOGOUS ARTERY CORONARY ARTERY BYPASS GRAFT(S) WITH ANGINA PECTORIS WITH DOCUMENTED SPASM |
| I25.728 | ATHEROSCLEROSIS OF AUTOLOGOUS ARTERY CORONARY ARTERY BYPASS GRAFT(S) WITH OTHER FORMS OF ANGINA PECTORIS |
| I25.729 | ATHEROSCLEROSIS OF AUTOLOGOUS ARTERY CORONARY ARTERY BYPASS GRAFT(S) WITH UNSPECIFIED ANGINA PECTORIS |
| I25.73 | ATHEROSCLEROSIS OF NONAUTOLOGOUS BIOLOGICAL CORONARY ARTERY BYPASS GRAFT(S) WITH ANGINA PECTORIS |
| I25.730 | ATHEROSCLEROSIS OF NONAUTOLOGOUS BIOLOGICAL CORONARY ARTERY BYPASS GRAFT(S) WITH UNSTABLE ANGINA PECTORIS |
| I25.731 | ATHEROSCLEROSIS OF NONAUTOLOGOUS BIOLOGICAL CORONARY ARTERY BYPASS GRAFT(S) WITH ANGINA PECTORIS WITH DOCUMENTED SPASM |
| I25.738 | ATHEROSCLEROSIS OF NONAUTOLOGOUS BIOLOGICAL CORONARY ARTERY BYPASS GRAFT(S) WITH OTHER FORMS OF ANGINA PECTORIS |
| I25.739 | ATHEROSCLEROSIS OF NONAUTOLOGOUS BIOLOGICAL CORONARY ARTERY BYPASS GRAFT(S) WITH UNSPECIFIED ANGINA PECTORIS |
| I25.75 | ATHEROSCLEROSIS OF NATIVE CORONARY ARTERY OF TRANSPLANTED HEART WITH ANGINA PECTORIS |
| I25.750 | ATHEROSCLEROSIS OF NATIVE CORONARY ARTERY OF TRANSPLANTED HEART WITH UNSTABLE ANGINA |
| I25.751 | ATHEROSCLEROSIS OF NATIVE CORONARY ARTERY OF TRANSPLANTED HEART WITH ANGINA PECTORIS WITH DOCUMENTED SPASM |
| I25.758 | ATHEROSCLEROSIS OF NATIVE CORONARY ARTERY OF TRANSPLANTED HEART WITH OTHER FORMS OF ANGINA PECTORIS |
| I25.759 | ATHEROSCLEROSIS OF NATIVE CORONARY ARTERY OF TRANSPLANTED HEART WITH UNSPECIFIED ANGINA PECTORIS |
| I25.76 | ATHEROSCLEROSIS OF BYPASS GRAFT OF CORONARY ARTERY OF TRANSPLANTED HEART WITH ANGINA PECTORIS |
| I25.760 | ATHEROSCLEROSIS OF BYPASS GRAFT OF CORONARY ARTERY OF TRANSPLANTED HEART WITH UNSTABLE ANGINA |
| I25.761 | ATHEROSCLEROSIS OF BYPASS GRAFT OF CORONARY ARTERY OF TRANSPLANTED HEART WITH ANGINA PECTORIS WITH DOCUMENTED SPASM |
| I25.768 | ATHEROSCLEROSIS OF BYPASS GRAFT OF CORONARY ARTERY OF TRANSPLANTED HEART WITH OTHER FORMS OF ANGINA PECTORIS |
| I25.769 | ATHEROSCLEROSIS OF BYPASS GRAFT OF CORONARY ARTERY OF TRANSPLANTED HEART WITH UNSPECIFIED ANGINA PECTORIS |
| I25.79 | ATHEROSCLEROSIS OF OTHER CORONARY ARTERY BYPASS GRAFT(S) WITH ANGINA PECTORIS |
| I25.790 | ATHEROSCLEROSIS OF OTHER CORONARY ARTERY BYPASS GRAFT(S) WITH UNSTABLE ANGINA PECTORIS |
| I25.791 | ATHEROSCLEROSIS OF OTHER CORONARY ARTERY BYPASS GRAFT(S) WITH ANGINA PECTORIS WITH DOCUMENTED SPASM |
| I25.798 | ATHEROSCLEROSIS OF OTHER CORONARY ARTERY BYPASS GRAFT(S) WITH OTHER FORMS OF ANGINA PECTORIS |
| I25.799 | ATHEROSCLEROSIS OF OTHER CORONARY ARTERY BYPASS GRAFT(S) WITH UNSPECIFIED ANGINA PECTORIS |
| I25.8 | OTHER FORMS OF CHRONIC ISCHEMIC HEART DISEASE |
| I25.81 | ATHEROSCLEROSIS OF OTHER CORONARY VESSELS WITHOUT ANGINA PECTORIS |
| I25.810 | ATHEROSCLEROSIS OF CORONARY ARTERY BYPASS GRAFT(S) WITHOUT ANGINA PECTORIS |
| I25.811 | ATHEROSCLEROSIS OF NATIVE CORONARY ARTERY OF TRANSPLANTED HEART WITHOUT ANGINA PECTORIS |
| I25.812 | ATHEROSCLEROSIS OF BYPASS GRAFT OF CORONARY ARTERY OF TRANSPLANTED HEART WITHOUT ANGINA PECTORIS |
| I25.82 | CHRONIC TOTAL OCCLUSION OF CORONARY ARTERY |
| I25.83 | CORONARY ATHEROSCLEROSIS DUE TO LIPID RICH PLAQUE |
| I25.84 | CORONARY ATHEROSCLEROSIS DUE TO CALCIFIED CORONARY LESION |
| I25.89 | OTHER FORMS OF CHRONIC ISCHEMIC HEART DISEASE |
| I25.9 | CHRONIC ISCHEMIC HEART DISEASE, UNSPECIFIED |
| Z95.1 | PRESENCE OF AORTOCORONARY BYPASS GRAFT |

S Table 2: Atherosclerotic Cardiovascular Disease ICD10 Codes for ischemic cerebrovascular disease

| ICD10 Code | Code Description |
| --- | --- |
| G45 | TRANSIENT CEREBRAL ISCHEMIC ATTACKS AND RELATED SYNDROMES |
| G45.0 | VERTEBRO-BASILAR ARTERY SYNDROME |
| G45.1 | CAROTID ARTERY SYNDROME (HEMISPHERIC) |
| G45.2 | MULTIPLE AND BILATERAL PRECEREBRAL ARTERY SYNDROMES |
| G45.3 | AMAUROSIS FUGAX |
| G45.4 | TRANSIENT GLOBAL AMNESIA |
| G45.8 | OTHER TRANSIENT CEREBRAL ISCHEMIC ATTACKS AND RELATED SYNDROMES |
| G45.9 | TRANSIENT CEREBRAL ISCHEMIC ATTACK, UNSPECIFIED |
| G46.0 | MIDDLE CEREBRAL ARTERY SYNDROME |
| G46.1 | ANTERIOR CEREBRAL ARTERY SYNDROME |
| G46.2 | POSTERIOR CEREBRAL ARTERY SYNDROME |
| G46.3 | BRAIN STEM STROKE SYNDROME |
| G46.4 | CEREBELLAR STROKE SYNDROME |
| G46.5 | PURE MOTOR LACUNAR SYNDROME |
| G46.6 | PURE SENSORY LACUNAR SYNDROME |
| G46.7 | OTHER LACUNAR SYNDROMES |
| I63 | CEREBRAL INFARCTION |
| I63.0 | CEREBRAL INFARCTION DUE TO THROMBOSIS OF PRECEREBRAL ARTERIES |
| I63.00 | CEREBRAL INFARCTION DUE TO THROMBOSIS OF UNSPECIFIED PRECEREBRAL ARTERY |
| I63.01 | CEREBRAL INFARCTION DUE TO THROMBOSIS OF VERTEBRAL ARTERY |
| I63.011 | CEREBRAL INFARCTION DUE TO THROMBOSIS OF RIGHT VERTEBRAL ARTERY |
| I63.012 | CEREBRAL INFARCTION DUE TO THROMBOSIS OF LEFT VERTEBRAL ARTERY |
| I63.013 | CEREBRAL INFARCTION DUE TO THROMBOSIS OF BILATERAL VERTEBRAL ARTERIES |
| I63.019 | CEREBRAL INFARCTION DUE TO THROMBOSIS OF UNSPECIFIED VERTEBRAL ARTERY |
| I63.02 | CEREBRAL INFARCTION DUE TO THROMBOSIS OF BASILAR ARTERY |
| I63.03 | CEREBRAL INFARCTION DUE TO THROMBOSIS OF CAROTID ARTERY |
| I63.031 | CEREBRAL INFARCTION DUE TO THROMBOSIS OF RIGHT CAROTID ARTERY |
| I63.032 | CEREBRAL INFARCTION DUE TO THROMBOSIS OF LEFT CAROTID ARTERY |
| I63.033 | CEREBRAL INFARCTION DUE TO THROMBOSIS OF BILATERAL CAROTID ARTERIES |
| I63.039 | CEREBRAL INFARCTION DUE TO THROMBOSIS OF UNSPECIFIED CAROTID ARTERY |
| I63.09 | CEREBRAL INFARCTION DUE TO THROMBOSIS OF OTHER PRECEREBRAL ARTERY |
| I63.1 | CEREBRAL INFARCTION DUE TO EMBOLISM OF PRECEREBRAL ARTERIES |
| I63.10 | CEREBRAL INFARCTION DUE TO EMBOLISM OF UNSPECIFIED PRECEREBRAL ARTERY |
| I63.11 | CEREBRAL INFARCTION DUE TO EMBOLISM OF VERTEBRAL ARTERY |
| I63.111 | CEREBRAL INFARCTION DUE TO EMBOLISM OF RIGHT VERTEBRAL ARTERY |
| I63.112 | CEREBRAL INFARCTION DUE TO EMBOLISM OF LEFT VERTEBRAL ARTERY |
| I63.113 | CEREBRAL INFARCTION DUE TO EMBOLISM OF BILATERAL VERTEBRAL ARTERIES |
| I63.119 | CEREBRAL INFARCTION DUE TO EMBOLISM OF UNSPECIFIED VERTEBRAL ARTERY |
| I63.12 | CEREBRAL INFARCTION DUE TO EMBOLISM OF BASILAR ARTERY |
| I63.13 | CEREBRAL INFARCTION DUE TO EMBOLISM OF CAROTID ARTERY |
| I63.131 | CEREBRAL INFARCTION DUE TO EMBOLISM OF RIGHT CAROTID ARTERY |
| I63.132 | CEREBRAL INFARCTION DUE TO EMBOLISM OF LEFT CAROTID ARTERY |
| I63.133 | CEREBRAL INFARCTION DUE TO EMBOLISM OF BILATERAL CAROTID ARTERIES |
| I63.139 | CEREBRAL INFARCTION DUE TO EMBOLISM OF UNSPECIFIED CAROTID ARTERY |
| I63.19 | CEREBRAL INFARCTION DUE TO EMBOLISM OF OTHER PRECEREBRAL ARTERY |
| I63.2 | CEREBRAL INFARCTION DUE TO UNSPECIFIED OCCLUSION OR STENOSIS OF PRECEREBRAL ARTERIES |
| I63.20 | CEREBRAL INFARCTION DUE TO UNSPECIFIED OCCLUSION OR STENOSIS OF UNSPECIFIED PRECEREBRAL ARTERIES |
| I63.21 | CEREBRAL INFARCTION DUE TO UNSPECIFIED OCCLUSION OR STENOSIS OF VERTEBRAL ARTERIES |
| I63.211 | CEREBRAL INFARCTION DUE TO UNSPECIFIED OCCLUSION OR STENOSIS OF RIGHT VERTEBRAL ARTERY |
| I63.212 | CEREBRAL INFARCTION DUE TO UNSPECIFIED OCCLUSION OR STENOSIS OF LEFT VERTEBRAL ARTERY |
| I63.213 | CEREBRAL INFARCTION DUE TO UNSPECIFIED OCCLUSION OR STENOSIS OF BILATERAL VERTEBRAL ARTERIES |
| I63.219 | CEREBRAL INFARCTION DUE TO UNSPECIFIED OCCLUSION OR STENOSIS OF UNSPECIFIED VERTEBRAL ARTERY |
| I63.22 | CEREBRAL INFARCTION DUE TO UNSPECIFIED OCCLUSION OR STENOSIS OF BASILAR ARTERY |
| I63.23 | CEREBRAL INFARCTION DUE TO UNSPECIFIED OCCLUSION OR STENOSIS OF CAROTID ARTERIES |
| I63.231 | CEREBRAL INFARCTION DUE TO UNSPECIFIED OCCLUSION OR STENOSIS OF RIGHT CAROTID ARTERIES |
| I63.232 | CEREBRAL INFARCTION DUE TO UNSPECIFIED OCCLUSION OR STENOSIS OF LEFT CAROTID ARTERIES |
| I63.233 | CEREBRAL INFARCTION DUE TO UNSPECIFIED OCCLUSION OR STENOSIS OF BILATERAL CAROTID ARTERIES |
| I63.239 | CEREBRAL INFARCTION DUE TO UNSPECIFIED OCCLUSION OR STENOSIS OF UNSPECIFIED CAROTID ARTERY |
| I63.29 | CEREBRAL INFARCTION DUE TO UNSPECIFIED OCCLUSION OR STENOSIS OF OTHER PRECEREBRAL ARTERIES |
| I63.3 | CEREBRAL INFARCTION DUE TO THROMBOSIS OF CEREBRAL ARTERIES |
| I63.30 | CEREBRAL INFARCTION DUE TO THROMBOSIS OF UNSPECIFIED CEREBRAL ARTERY |
| I63.31 | CEREBRAL INFARCTION DUE TO THROMBOSIS OF MIDDLE CEREBRAL ARTERY |
| I63.311 | CEREBRAL INFARCTION DUE TO THROMBOSIS OF RIGHT MIDDLE CEREBRAL ARTERY |
| I63.312 | CEREBRAL INFARCTION DUE TO THROMBOSIS OF LEFT MIDDLE CEREBRAL ARTERY |
| I63.313 | CEREBRAL INFARCTION DUE TO THROMBOSIS OF BILATERAL MIDDLE CEREBRAL ARTERIES |
| I63.319 | CEREBRAL INFARCTION DUE TO THROMBOSIS OF UNSPECIFIED MIDDLE CEREBRAL ARTERY |
| I63.32 | CEREBRAL INFARCTION DUE TO THROMBOSIS OF ANTERIOR CEREBRAL ARTERY |
| I63.321 | CEREBRAL INFARCTION DUE TO THROMBOSIS OF RIGHT ANTERIOR CEREBRAL ARTERY |
| I63.322 | CEREBRAL INFARCTION DUE TO THROMBOSIS OF LEFT ANTERIOR CEREBRAL ARTERY |
| I63.323 | CEREBRAL INFARCTION DUE TO THROMBOSIS OF BILATERAL ANTERIOR CEREBRAL ARTERIES |
| I63.329 | CEREBRAL INFARCTION DUE TO THROMBOSIS OF UNSPECIFIED ANTERIOR CEREBRAL ARTERY |
| I63.33 | CEREBRAL INFARCTION DUE TO THROMBOSIS OF POSTERIOR CEREBRAL ARTERY |
| I63.331 | CEREBRAL INFARCTION DUE TO THROMBOSIS OF RIGHT POSTERIOR CEREBRAL ARTERY |
| I63.332 | CEREBRAL INFARCTION DUE TO THROMBOSIS OF LEFT POSTERIOR CEREBRAL ARTERY |
| I63.333 | CEREBRAL INFARCTION DUE TO THROMBOSIS OF BILATERAL POSTERIOR CEREBRAL ARTERIES |
| I63.339 | CEREBRAL INFARCTION DUE TO THROMBOSIS OF UNSPECIFIED POSTERIOR CEREBRAL ARTERY |
| I63.34 | CEREBRAL INFARCTION DUE TO THROMBOSIS OF CEREBELLAR ARTERY |
| I63.341 | CEREBRAL INFARCTION DUE TO THROMBOSIS OF RIGHT CEREBELLAR ARTERY |
| I63.342 | CEREBRAL INFARCTION DUE TO THROMBOSIS OF LEFT CEREBELLAR ARTERY |
| I63.343 | CEREBRAL INFARCTION DUE TO THROMBOSIS OF BILATERAL CEREBELLAR ARTERIES |
| I63.349 | CEREBRAL INFARCTION DUE TO THROMBOSIS OF UNSPECIFIED CEREBELLAR ARTERY |
| I63.39 | CEREBRAL INFARCTION DUE TO THROMBOSIS OF OTHER CEREBRAL ARTERY |
| I63.4 | CEREBRAL INFARCTION DUE TO EMBOLISM OF CEREBRAL ARTERIES |
| I63.40 | CEREBRAL INFARCTION DUE TO EMBOLISM OF UNSPECIFIED CEREBRAL ARTERY |
| I63.41 | CEREBRAL INFARCTION DUE TO EMBOLISM OF MIDDLE CEREBRAL ARTERY |
| I63.411 | CEREBRAL INFARCTION DUE TO EMBOLISM OF RIGHT MIDDLE CEREBRAL ARTERY |
| I63.412 | CEREBRAL INFARCTION DUE TO EMBOLISM OF LEFT MIDDLE CEREBRAL ARTERY |
| I63.413 | CEREBRAL INFARCTION DUE TO EMBOLISM OF BILATERAL MIDDLE CEREBRAL ARTERIES |
| I63.419 | CEREBRAL INFARCTION DUE TO EMBOLISM OF UNSPECIFIED MIDDLE CEREBRAL ARTERY |
| I63.42 | CEREBRAL INFARCTION DUE TO EMBOLISM OF ANTERIOR CEREBRAL ARTERY |
| I63.421 | CEREBRAL INFARCTION DUE TO EMBOLISM OF RIGHT ANTERIOR CEREBRAL ARTERY |
| I63.422 | CEREBRAL INFARCTION DUE TO EMBOLISM OF LEFT ANTERIOR CEREBRAL ARTERY |
| I63.423 | CEREBRAL INFARCTION DUE TO EMBOLISM OF BILATERAL ANTERIOR CEREBRAL ARTERIES |
| I63.429 | CEREBRAL INFARCTION DUE TO EMBOLISM OF UNSPECIFIED ANTERIOR CEREBRAL ARTERY |
| I63.43 | CEREBRAL INFARCTION DUE TO EMBOLISM OF POSTERIOR CEREBRAL ARTERY |
| I63.431 | CEREBRAL INFARCTION DUE TO EMBOLISM OF RIGHT POSTERIOR CEREBRAL ARTERY |
| I63.432 | CEREBRAL INFARCTION DUE TO EMBOLISM OF LEFT POSTERIOR CEREBRAL ARTERY |
| I63.433 | CEREBRAL INFARCTION DUE TO EMBOLISM OF BILATERAL POSTERIOR CEREBRAL ARTERIES |
| I63.439 | CEREBRAL INFARCTION DUE TO EMBOLISM OF UNSPECIFIED POSTERIOR CEREBRAL ARTERY |
| I63.44 | CEREBRAL INFARCTION DUE TO EMBOLISM OF CEREBELLAR ARTERY |
| I63.441 | CEREBRAL INFARCTION DUE TO EMBOLISM OF RIGHT CEREBELLAR ARTERY |
| I63.442 | CEREBRAL INFARCTION DUE TO EMBOLISM OF LEFT CEREBELLAR ARTERY |
| I63.443 | CEREBRAL INFARCTION DUE TO EMBOLISM OF BILATERAL CEREBELLAR ARTERIES |
| I63.449 | CEREBRAL INFARCTION DUE TO EMBOLISM OF UNSPECIFIED CEREBELLAR ARTERY |
| I63.49 | CEREBRAL INFARCTION DUE TO EMBOLISM OF OTHER CEREBRAL ARTERY |
| I63.5 | CEREBRAL INFARCTION DUE TO UNSPECIFIED OCCLUSION OR STENOSIS OF CEREBRAL ARTERIES |
| I63.50 | CEREBRAL INFARCTION DUE TO UNSPECIFIED OCCLUSION OR STENOSIS OF UNSPECIFIED CEREBRAL ARTERY |
| I63.51 | CEREBRAL INFARCTION DUE TO UNSPECIFIED OCCLUSION OR STENOSIS OF MIDDLE CEREBRAL ARTERY |
| I63.511 | CEREBRAL INFARCTION DUE TO UNSPECIFIED OCCLUSION OR STENOSIS OF RIGHT MIDDLE CEREBRAL ARTERY |
| I63.512 | CEREBRAL INFARCTION DUE TO UNSPECIFIED OCCLUSION OR STENOSIS OF LEFT MIDDLE CEREBRAL ARTERY |
| I63.513 | CEREBRAL INFARCTION DUE TO UNSPECIFIED OCCLUSION OR STENOSIS OF BILATERAL MIDDLE CEREBRAL ARTERIES |
| I63.519 | CEREBRAL INFARCTION DUE TO UNSPECIFIED OCCLUSION OR STENOSIS OF UNSPECIFIED MIDDLE CEREBRAL ARTERY |
| I63.52 | CEREBRAL INFARCTION DUE TO UNSPECIFIED OCCLUSION OR STENOSIS OF ANTERIOR CEREBRAL ARTERY |
| I63.521 | CEREBRAL INFARCTION DUE TO UNSPECIFIED OCCLUSION OR STENOSIS OF RIGHT ANTERIOR CEREBRAL ARTERY |
| I63.522 | CEREBRAL INFARCTION DUE TO UNSPECIFIED OCCLUSION OR STENOSIS OF LEFT ANTERIOR CEREBRAL ARTERY |
| I63.523 | CEREBRAL INFARCTION DUE TO UNSPECIFIED OCCLUSION OR STENOSIS OF BILATERAL ANTERIOR CEREBRAL ARTERIES |
| I63.529 | CEREBRAL INFARCTION DUE TO UNSPECIFIED OCCLUSION OR STENOSIS OF UNSPECIFIED ANTERIOR CEREBRAL ARTERY |
| I63.53 | CEREBRAL INFARCTION DUE TO UNSPECIFIED OCCLUSION OR STENOSIS OF POSTERIOR CEREBRAL ARTERY |
| I63.531 | CEREBRAL INFARCTION DUE TO UNSPECIFIED OCCLUSION OR STENOSIS OF RIGHT POSTERIOR CEREBRAL ARTERY |
| I63.532 | CEREBRAL INFARCTION DUE TO UNSPECIFIED OCCLUSION OR STENOSIS OF LEFT POSTERIOR CEREBRAL ARTERY |
| I63.533 | CEREBRAL INFARCTION DUE TO UNSPECIFIED OCCLUSION OR STENOSIS OF BILATERAL POSTERIOR CEREBRAL ARTERIES |
| I63.539 | CEREBRAL INFARCTION DUE TO UNSPECIFIED OCCLUSION OR STENOSIS OF UNSPECIFIED POSTERIOR CEREBRAL ARTERY |
| I63.54 | CEREBRAL INFARCTION DUE TO UNSPECIFIED OCCLUSION OR STENOSIS OF CEREBELLAR ARTERY |
| I63.541 | CEREBRAL INFARCTION DUE TO UNSPECIFIED OCCLUSION OR STENOSIS OF RIGHT CEREBELLAR ARTERY |
| I63.542 | CEREBRAL INFARCTION DUE TO UNSPECIFIED OCCLUSION OR STENOSIS OF LEFT CEREBELLAR ARTERY |
| I63.543 | CEREBRAL INFARCTION DUE TO UNSPECIFIED OCCLUSION OR STENOSIS OF BILATERAL CEREBELLAR ARTERIES |
| I63.549 | CEREBRAL INFARCTION DUE TO UNSPECIFIED OCCLUSION OR STENOSIS OF UNSPECIFIED CEREBELLAR ARTERY |
| I63.59 | CEREBRAL INFARCTION DUE TO UNSPECIFIED OCCLUSION OR STENOSIS OF OTHER CEREBRAL ARTERY |
| I63.6 | CEREBRAL INFARCTION DUE TO CEREBRAL VENOUS THROMBOSIS, NONPYOGENIC |
| I63.8 | OTHER CEREBRAL INFARCTION |
| I63.81 | OTHER CEREBRAL INFARCTION DUE TO OCCLUSION OR STENOSIS OF SMALL ARTERY |
| I63.89 | OTHER CEREBRAL INFARCTION |
| I63.9 | CEREBRAL INFARCTION, UNSPECIFIED |
| I65 | OCCLUSION AND STENOSIS OF PRECEREBRAL ARTERIES, NOT RESULTING IN CEREBRAL INFARCTION |
| I65.0 | OCCLUSION AND STENOSIS OF VERTEBRAL ARTERY |
| I65.01 | OCCLUSION AND STENOSIS OF RIGHT VERTEBRAL ARTERY |
| I65.02 | OCCLUSION AND STENOSIS OF LEFT VERTEBRAL ARTERY |
| I65.03 | OCCLUSION AND STENOSIS OF BILATERAL VERTEBRAL ARTERIES |
| I65.09 | OCCLUSION AND STENOSIS OF UNSPECIFIED VERTEBRAL ARTERY |
| I65.1 | OCCLUSION AND STENOSIS OF BASILAR ARTERY |
| I65.2 | OCCLUSION AND STENOSIS OF CAROTID ARTERY |
| I65.21 | OCCLUSION AND STENOSIS OF RIGHT CAROTID ARTERY |
| I65.22 | OCCLUSION AND STENOSIS OF LEFT CAROTID ARTERY |
| I65.23 | OCCLUSION AND STENOSIS OF BILATERAL CAROTID ARTERIES |
| I65.29 | OCCLUSION AND STENOSIS OF UNSPECIFIED CAROTID ARTERY |
| I65.8 | OCCLUSION AND STENOSIS OF OTHER PRECEREBRAL ARTERIES |
| I65.9 | OCCLUSION AND STENOSIS OF UNSPECIFIED PRECEREBRAL ARTERY |
| I66 | OCCLUSION AND STENOSIS OF CEREBRAL ARTERIES, NOT RESULTING IN CEREBRAL INFARCTION |
| I66.0 | OCCLUSION AND STENOSIS OF MIDDLE CEREBRAL ARTERY |
| I66.01 | OCCLUSION AND STENOSIS OF RIGHT MIDDLE CEREBRAL ARTERY |
| I66.02 | OCCLUSION AND STENOSIS OF LEFT MIDDLE CEREBRAL ARTERY |
| I66.03 | OCCLUSION AND STENOSIS OF BILATERAL MIDDLE CEREBRAL ARTERIES |
| I66.09 | OCCLUSION AND STENOSIS OF UNSPECIFIED MIDDLE CEREBRAL ARTERY |
| I66.1 | OCCLUSION AND STENOSIS OF ANTERIOR CEREBRAL ARTERY |
| I66.11 | OCCLUSION AND STENOSIS OF RIGHT ANTERIOR CEREBRAL ARTERY |
| I66.12 | OCCLUSION AND STENOSIS OF LEFT ANTERIOR CEREBRAL ARTERY |
| I66.13 | OCCLUSION AND STENOSIS OF BILATERAL ANTERIOR CEREBRAL ARTERIES |
| I66.19 | OCCLUSION AND STENOSIS OF UNSPECIFIED ANTERIOR CEREBRAL ARTERY |
| I66.2 | OCCLUSION AND STENOSIS OF POSTERIOR CEREBRAL ARTERY |
| I66.21 | OCCLUSION AND STENOSIS OF RIGHT POSTERIOR CEREBRAL ARTERY |
| I66.22 | OCCLUSION AND STENOSIS OF LEFT POSTERIOR CEREBRAL ARTERY |
| I66.23 | OCCLUSION AND STENOSIS OF BILATERAL POSTERIOR CEREBRAL ARTERIES |
| I66.29 | OCCLUSION AND STENOSIS OF UNSPECIFIED POSTERIOR CEREBRAL ARTERY |
| I66.3 | OCCLUSION AND STENOSIS OF CEREBELLAR ARTERIES |
| I66.8 | OCCLUSION AND STENOSIS OF OTHER CEREBRAL ARTERIES |
| I66.9 | OCCLUSION AND STENOSIS OF UNSPECIFIED CEREBRAL ARTERY |
| I67.2 | CEREBRAL ATHEROSCLEROSIS |
| I67.81 | ACUTE CEREBROVASCULAR INSUFFICIENCY |
| I67.82 | CEREBRAL ISCHEMIA |
| I69.3 | SEQUELAE OF CEREBRAL INFARCTION |
| I69.30 | UNSPECIFIED SEQUELAE OF CEREBRAL INFARCTION |
| I69.31 | COGNITIVE DEFICITS FOLLOWING CEREBRAL INFARCTION |
| I69.310 | ATTENTION AND CONCENTRATION DEFICIT FOLLOWING CEREBRAL INFARCTION |
| I69.311 | MEMORY DEFICIT FOLLOWING CEREBRAL INFARCTION |
| I69.312 | VISUOSPATIAL DEFICIT AND SPATIAL NEGLECT FOLLOWING CEREBRAL INFARCTION |
| I69.313 | PSYCHOMOTOR DEFICIT FOLLOWING CEREBRAL INFARCTION |
| I69.314 | FRONTAL LOBE AND EXECUTIVE FUNCTION DEFICIT FOLLOWING CEREBRAL INFARCTION |
| I69.315 | COGNITIVE SOCIAL OR EMOTIONAL DEFICIT FOLLOWING CEREBRAL INFARCTION |
| I69.318 | OTHER SYMPTOMS AND SIGNS INVOLVING COGNITIVE FUNCTIONS FOLLOWING CEREBRAL INFARCTION |
| I69.319 | UNSPECIFIED SYMPTOMS AND SIGNS INVOLVING COGNITIVE FUNCTIONS FOLLOWING CEREBRAL INFARCTION |
| I69.32 | SPEECH AND LANGUAGE DEFICITS FOLLOWING CEREBRAL INFARCTION |
| I69.320 | APHASIA FOLLOWING CEREBRAL INFARCTION |
| I69.321 | DYSPHASIA FOLLOWING CEREBRAL INFARCTION |
| I69.322 | DYSARTHRIA FOLLOWING CEREBRAL INFARCTION |
| I69.323 | FLUENCY DISORDER FOLLOWING CEREBRAL INFARCTION |
| I69.328 | OTHER SPEECH AND LANGUAGE DEFICITS FOLLOWING CEREBRAL INFARCTION |
| I69.33 | MONOPLEGIA OF UPPER LIMB FOLLOWING CEREBRAL INFARCTION |
| I69.331 | MONOPLEGIA OF UPPER LIMB FOLLOWING CEREBRAL INFARCTION AFFECTING RIGHT DOMINANT SIDE |
| I69.332 | MONOPLEGIA OF UPPER LIMB FOLLOWING CEREBRAL INFARCTION AFFECTING LEFT DOMINANT SIDE |
| I69.333 | MONOPLEGIA OF UPPER LIMB FOLLOWING CEREBRAL INFARCTION AFFECTING RIGHT NON-DOMINANT SIDE |
| I69.334 | MONOPLEGIA OF UPPER LIMB FOLLOWING CEREBRAL INFARCTION AFFECTING LEFT NON-DOMINANT SIDE |
| I69.339 | MONOPLEGIA OF UPPER LIMB FOLLOWING CEREBRAL INFARCTION AFFECTING UNSPECIFIED SIDE |
| I69.34 | MONOPLEGIA OF LOWER LIMB FOLLOWING CEREBRAL INFARCTION |
| I69.341 | MONOPLEGIA OF LOWER LIMB FOLLOWING CEREBRAL INFARCTION AFFECTING RIGHT DOMINANT SIDE |
| I69.342 | MONOPLEGIA OF LOWER LIMB FOLLOWING CEREBRAL INFARCTION AFFECTING LEFT DOMINANT SIDE |
| I69.343 | MONOPLEGIA OF LOWER LIMB FOLLOWING CEREBRAL INFARCTION AFFECTING RIGHT NON-DOMINANT SIDE |
| I69.344 | MONOPLEGIA OF LOWER LIMB FOLLOWING CEREBRAL INFARCTION AFFECTING LEFT NON-DOMINANT SIDE |
| I69.349 | MONOPLEGIA OF LOWER LIMB FOLLOWING CEREBRAL INFARCTION AFFECTING UNSPECIFIED SIDE |
| I69.35 | HEMIPLEGIA AND HEMIPARESIS FOLLOWING CEREBRAL INFARCTION |
| I69.351 | HEMIPLEGIA AND HEMIPARESIS FOLLOWING CEREBRAL INFARCTION AFFECTING RIGHT DOMINANT SIDE |
| I69.352 | HEMIPLEGIA AND HEMIPARESIS FOLLOWING CEREBRAL INFARCTION AFFECTING LEFT DOMINANT SIDE |
| I69.353 | HEMIPLEGIA AND HEMIPARESIS FOLLOWING CEREBRAL INFARCTION AFFECTING RIGHT NON-DOMINANT SIDE |
| I69.354 | HEMIPLEGIA AND HEMIPARESIS FOLLOWING CEREBRAL INFARCTION AFFECTING LEFT NON-DOMINANT SIDE |
| I69.359 | HEMIPLEGIA AND HEMIPARESIS FOLLOWING CEREBRAL INFARCTION AFFECTING UNSPECIFIED SIDE |
| I69.36 | OTHER PARALYTIC SYNDROME FOLLOWING CEREBRAL INFARCTION |
| I69.361 | OTHER PARALYTIC SYNDROME FOLLOWING CEREBRAL INFARCTION AFFECTING RIGHT DOMINANT SIDE |
| I69.362 | OTHER PARALYTIC SYNDROME FOLLOWING CEREBRAL INFARCTION AFFECTING LEFT DOMINANT SIDE |
| I69.363 | OTHER PARALYTIC SYNDROME FOLLOWING CEREBRAL INFARCTION AFFECTING RIGHT NON-DOMINANT SIDE |
| I69.364 | OTHER PARALYTIC SYNDROME FOLLOWING CEREBRAL INFARCTION AFFECTING LEFT NON-DOMINANT SIDE |
| I69.365 | OTHER PARALYTIC SYNDROME FOLLOWING CEREBRAL INFARCTION, BILATERAL |
| I69.369 | OTHER PARALYTIC SYNDROME FOLLOWING CEREBRAL INFARCTION AFFECTING UNSPECIFIED SIDE |
| I69.39 | OTHER SEQUELAE OF CEREBRAL INFARCTION |
| I69.390 | APRAXIA FOLLOWING CEREBRAL INFARCTION |
| I69.391 | DYSPHAGIA FOLLOWING CEREBRAL INFARCTION |
| I69.392 | FACIAL WEAKNESS FOLLOWING CEREBRAL INFARCTION |
| I69.393 | ATAXIA FOLLOWING CEREBRAL INFARCTION |
| I69.398 | OTHER SEQUELAE OF CEREBRAL INFARCTION |
| I69.8 | SEQUELAE OF OTHER CEREBROVASCULAR DISEASES |

STable 3: Atherosclerotic Cardiovascular Disease ICD10 Codes for peripheral arterial disease

| ICD10 Code | Code Description |
| --- | --- |
| I70.0 | ATHEROSCLEROSIS OF AORTA |
| I70.1 | ATHEROSCLEROSIS OF RENAL ARTERY |
| I70.2 | ATHEROSCLEROSIS OF NATIVE ARTERIES OF THE EXTREMITIES |
| I70.20 | UNSPECIFIED ATHEROSCLEROSIS OF NATIVE ARTERIES OF EXTREMITIES |
| I70.201 | UNSPECIFIED ATHEROSCLEROSIS OF NATIVE ARTERIES OF EXTREMITIES, RIGHT LEG |
| I70.202 | UNSPECIFIED ATHEROSCLEROSIS OF NATIVE ARTERIES OF EXTREMITIES, LEFT LEG |
| I70.203 | UNSPECIFIED ATHEROSCLEROSIS OF NATIVE ARTERIES OF EXTREMITIES, BILATERAL LEGS |
| I70.208 | UNSPECIFIED ATHEROSCLEROSIS OF NATIVE ARTERIES OF EXTREMITIES, OTHER EXTREMITY |
| I70.209 | UNSPECIFIED ATHEROSCLEROSIS OF NATIVE ARTERIES OF EXTREMITIES, UNSPECIFIED EXTREMITY |
| I70.21 | ATHEROSCLEROSIS OF NATIVE ARTERIES OF EXTREMITIES WITH INTERMITTENT CLAUDICATION |
| I70.211 | ATHEROSCLEROSIS OF NATIVE ARTERIES OF EXTREMITIES WITH INTERMITTENT CLAUDICATION, RIGHT LEG |
| I70.212 | ATHEROSCLEROSIS OF NATIVE ARTERIES OF EXTREMITIES WITH INTERMITTENT CLAUDICATION, LEFT LEG |
| I70.213 | ATHEROSCLEROSIS OF NATIVE ARTERIES OF EXTREMITIES WITH INTERMITTENT CLAUDICATION, BILATERAL LEGS |
| I70.218 | ATHEROSCLEROSIS OF NATIVE ARTERIES OF EXTREMITIES WITH INTERMITTENT CLAUDICATION, OTHER EXTREMITY |
| I70.219 | ATHEROSCLEROSIS OF NATIVE ARTERIES OF EXTREMITIES WITH INTERMITTENT CLAUDICATION, UNSPECIFIED EXTREMITY |
| I70.22 | ATHEROSCLEROSIS OF NATIVE ARTERIES OF EXTREMITIES WITH REST PAIN |
| I70.221 | ATHEROSCLEROSIS OF NATIVE ARTERIES OF EXTREMITIES WITH REST PAIN, RIGHT LEG |
| I70.222 | ATHEROSCLEROSIS OF NATIVE ARTERIES OF EXTREMITIES WITH REST PAIN, LEFT LEG |
| I70.223 | ATHEROSCLEROSIS OF NATIVE ARTERIES OF EXTREMITIES WITH REST PAIN, BILATERAL LEGS |
| I70.228 | ATHEROSCLEROSIS OF NATIVE ARTERIES OF EXTREMITIES WITH REST PAIN, OTHER EXTREMITY |
| I70.229 | ATHEROSCLEROSIS OF NATIVE ARTERIES OF EXTREMITIES WITH REST PAIN, UNSPECIFIED EXTREMITY |
| I70.23 | ATHEROSCLEROSIS OF NATIVE ARTERIES OF RIGHT LEG WITH ULCERATION |
| I70.231 | ATHEROSCLEROSIS OF NATIVE ARTERIES OF RIGHT LEG WITH ULCERATION OF THIGH |
| I70.232 | ATHEROSCLEROSIS OF NATIVE ARTERIES OF RIGHT LEG WITH ULCERATION OF CALF |
| I70.233 | ATHEROSCLEROSIS OF NATIVE ARTERIES OF RIGHT LEG WITH ULCERATION OF ANKLE |
| I70.234 | ATHEROSCLEROSIS OF NATIVE ARTERIES OF RIGHT LEG WITH ULCERATION OF HEEL AND MIDFOOT |
| I70.235 | ATHEROSCLEROSIS OF NATIVE ARTERIES OF RIGHT LEG WITH ULCERATION OF OTHER PART OF FOOT |
| I70.238 | ATHEROSCLEROSIS OF NATIVE ARTERIES OF RIGHT LEG WITH ULCERATION OF OTHER PART OF LOWER LEG |
| I70.239 | ATHEROSCLEROSIS OF NATIVE ARTERIES OF RIGHT LEG WITH ULCERATION OF UNSPECIFIED SITE |
| I70.24 | ATHEROSCLEROSIS OF NATIVE ARTERIES OF LEFT LEG WITH ULCERATION |
| I70.241 | ATHEROSCLEROSIS OF NATIVE ARTERIES OF LEFT LEG WITH ULCERATION OF THIGH |
| I70.242 | ATHEROSCLEROSIS OF NATIVE ARTERIES OF LEFT LEG WITH ULCERATION OF CALF |
| I70.243 | ATHEROSCLEROSIS OF NATIVE ARTERIES OF LEFT LEG WITH ULCERATION OF ANKLE |
| I70.244 | ATHEROSCLEROSIS OF NATIVE ARTERIES OF LEFT LEG WITH ULCERATION OF HEEL AND MIDFOOT |
| I70.245 | ATHEROSCLEROSIS OF NATIVE ARTERIES OF LEFT LEG WITH ULCERATION OF OTHER PART OF FOOT |
| I70.248 | ATHEROSCLEROSIS OF NATIVE ARTERIES OF LEFT LEG WITH ULCERATION OF OTHER PART OF LOWER LEG |
| I70.249 | ATHEROSCLEROSIS OF NATIVE ARTERIES OF LEFT LEG WITH ULCERATION OF UNSPECIFIED SITE |
| I70.25 | ATHEROSCLEROSIS OF NATIVE ARTERIES OF OTHER EXTREMITIES WITH ULCERATION |
| I70.26 | ATHEROSCLEROSIS OF NATIVE ARTERIES OF EXTREMITIES WITH GANGRENE |
| I70.261 | ATHEROSCLEROSIS OF NATIVE ARTERIES OF EXTREMITIES WITH GANGRENE, RIGHT LEG |
| I70.262 | ATHEROSCLEROSIS OF NATIVE ARTERIES OF EXTREMITIES WITH GANGRENE, LEFT LEG |
| I70.263 | ATHEROSCLEROSIS OF NATIVE ARTERIES OF EXTREMITIES WITH GANGRENE, BILATERAL LEGS |
| I70.268 | ATHEROSCLEROSIS OF NATIVE ARTERIES OF EXTREMITIES WITH GANGRENE, OTHER EXTREMITY |
| I70.269 | ATHEROSCLEROSIS OF NATIVE ARTERIES OF EXTREMITIES WITH GANGRENE, UNSPECIFIED EXTREMITY |
| I70.29 | OTHER ATHEROSCLEROSIS OF NATIVE ARTERIES OF EXTREMITIES |
| I70.291 | OTHER ATHEROSCLEROSIS OF NATIVE ARTERIES OF EXTREMITIES, RIGHT LEG |
| I70.292 | OTHER ATHEROSCLEROSIS OF NATIVE ARTERIES OF EXTREMITIES, LEFT LEG |
| I70.293 | OTHER ATHEROSCLEROSIS OF NATIVE ARTERIES OF EXTREMITIES, BILATERAL LEGS |
| I70.298 | OTHER ATHEROSCLEROSIS OF NATIVE ARTERIES OF EXTREMITIES, OTHER EXTREMITY |
| I70.299 | OTHER ATHEROSCLEROSIS OF NATIVE ARTERIES OF EXTREMITIES, UNSPECIFIED EXTREMITY |
| I70.3 | ATHEROSCLEROSIS OF UNSPECIFIED TYPE OF BYPASS GRAFT(S) OF THE EXTREMITIES |
| I70.30 | UNSPECIFIED ATHEROSCLEROSIS OF UNSPECIFIED TYPE OF BYPASS GRAFT(S) OF THE EXTREMITIES |
| I70.301 | UNSPECIFIED ATHEROSCLEROSIS OF UNSPECIFIED TYPE OF BYPASS GRAFT(S) OF THE EXTREMITIES, RIGHT LEG |
| I70.302 | UNSPECIFIED ATHEROSCLEROSIS OF UNSPECIFIED TYPE OF BYPASS GRAFT(S) OF THE EXTREMITIES, LEFT LEG |
| I70.303 | UNSPECIFIED ATHEROSCLEROSIS OF UNSPECIFIED TYPE OF BYPASS GRAFT(S) OF THE EXTREMITIES, BILATERAL LEGS |
| I70.308 | UNSPECIFIED ATHEROSCLEROSIS OF UNSPECIFIED TYPE OF BYPASS GRAFT(S) OF THE EXTREMITIES, OTHER EXTREMITY |
| I70.309 | UNSPECIFIED ATHEROSCLEROSIS OF UNSPECIFIED TYPE OF BYPASS GRAFT(S) OF THE EXTREMITIES, UNSPECIFIED EXTREMITY |
| I70.31 | ATHEROSCLEROSIS OF UNSPECIFIED TYPE OF BYPASS GRAFT(S) OF THE EXTREMITIES WITH INTERMITTENT CLAUDICATION |
| I70.311 | ATHEROSCLEROSIS OF UNSPECIFIED TYPE OF BYPASS GRAFT(S) OF THE EXTREMITIES WITH INTERMITTENT CLAUDICATION, RIGHT LEG |
| I70.312 | ATHEROSCLEROSIS OF UNSPECIFIED TYPE OF BYPASS GRAFT(S) OF THE EXTREMITIES WITH INTERMITTENT CLAUDICATION, LEFT LEG |
| I70.313 | ATHEROSCLEROSIS OF UNSPECIFIED TYPE OF BYPASS GRAFT(S) OF THE EXTREMITIES WITH INTERMITTENT CLAUDICATION, BILATERAL LEGS |
| I70.318 | ATHEROSCLEROSIS OF UNSPECIFIED TYPE OF BYPASS GRAFT(S) OF THE EXTREMITIES WITH INTERMITTENT CLAUDICATION, OTHER EXTREMITY |
| I70.319 | ATHEROSCLEROSIS OF UNSPECIFIED TYPE OF BYPASS GRAFT(S) OF THE EXTREMITIES WITH INTERMITTENT CLAUDICATION, UNSPECIFIED EXTREMITY |
| I70.32 | ATHEROSCLEROSIS OF UNSPECIFIED TYPE OF BYPASS GRAFT(S) OF THE EXTREMITIES WITH REST PAIN |
| I70.321 | ATHEROSCLEROSIS OF UNSPECIFIED TYPE OF BYPASS GRAFT(S) OF THE EXTREMITIES WITH REST PAIN, RIGHT LEG |
| I70.322 | ATHEROSCLEROSIS OF UNSPECIFIED TYPE OF BYPASS GRAFT(S) OF THE EXTREMITIES WITH REST PAIN, LEFT LEG |
| I70.323 | ATHEROSCLEROSIS OF UNSPECIFIED TYPE OF BYPASS GRAFT(S) OF THE EXTREMITIES WITH REST PAIN, BILATERAL LEGS |
| I70.328 | ATHEROSCLEROSIS OF UNSPECIFIED TYPE OF BYPASS GRAFT(S) OF THE EXTREMITIES WITH REST PAIN, OTHER EXTREMITY |
| I70.329 | ATHEROSCLEROSIS OF UNSPECIFIED TYPE OF BYPASS GRAFT(S) OF THE EXTREMITIES WITH REST PAIN, UNSPECIFIED EXTREMITY |
| I70.33 | ATHEROSCLEROSIS OF UNSPECIFIED TYPE OF BYPASS GRAFT(S) OF THE RIGHT LEG WITH ULCERATION |
| I70.331 | ATHEROSCLEROSIS OF UNSPECIFIED TYPE OF BYPASS GRAFT(S) OF THE RIGHT LEG WITH ULCERATION OF THIGH |
| I70.332 | ATHEROSCLEROSIS OF UNSPECIFIED TYPE OF BYPASS GRAFT(S) OF THE RIGHT LEG WITH ULCERATION OF CALF |
| I70.333 | ATHEROSCLEROSIS OF UNSPECIFIED TYPE OF BYPASS GRAFT(S) OF THE RIGHT LEG WITH ULCERATION OF ANKLE |
| I70.334 | ATHEROSCLEROSIS OF UNSPECIFIED TYPE OF BYPASS GRAFT(S) OF THE RIGHT LEG WITH ULCERATION OF HEEL AND MIDFOOT |
| I70.335 | ATHEROSCLEROSIS OF UNSPECIFIED TYPE OF BYPASS GRAFT(S) OF THE RIGHT LEG WITH ULCERATION OF OTHER PART OF FOOT |
| I70.338 | ATHEROSCLEROSIS OF UNSPECIFIED TYPE OF BYPASS GRAFT(S) OF THE RIGHT LEG WITH ULCERATION OF OTHER PART OF LOWER LEG |
| I70.339 | ATHEROSCLEROSIS OF UNSPECIFIED TYPE OF BYPASS GRAFT(S) OF THE RIGHT LEG WITH ULCERATION OF UNSPECIFIED SITE |
| I70.34 | ATHEROSCLEROSIS OF UNSPECIFIED TYPE OF BYPASS GRAFT(S) OF THE LEFT LEG WITH ULCERATION |
| I70.341 | ATHEROSCLEROSIS OF UNSPECIFIED TYPE OF BYPASS GRAFT(S) OF THE LEFT LEG WITH ULCERATION OF THIGH |
| I70.342 | ATHEROSCLEROSIS OF UNSPECIFIED TYPE OF BYPASS GRAFT(S) OF THE LEFT LEG WITH ULCERATION OF CALF |
| I70.343 | ATHEROSCLEROSIS OF UNSPECIFIED TYPE OF BYPASS GRAFT(S) OF THE LEFT LEG WITH ULCERATION OF ANKLE |
| I70.344 | ATHEROSCLEROSIS OF UNSPECIFIED TYPE OF BYPASS GRAFT(S) OF THE LEFT LEG WITH ULCERATION OF HEEL AND MIDFOOT |
| I70.345 | ATHEROSCLEROSIS OF UNSPECIFIED TYPE OF BYPASS GRAFT(S) OF THE LEFT LEG WITH ULCERATION OF OTHER PART OF FOOT |
| I70.348 | ATHEROSCLEROSIS OF UNSPECIFIED TYPE OF BYPASS GRAFT(S) OF THE LEFT LEG WITH ULCERATION OF OTHER PART OF LOWER LEG |
| I70.349 | ATHEROSCLEROSIS OF UNSPECIFIED TYPE OF BYPASS GRAFT(S) OF THE LEFT LEG WITH ULCERATION OF UNSPECIFIED SITE |
| I70.35 | ATHEROSCLEROSIS OF UNSPECIFIED TYPE OF BYPASS GRAFT(S) OF OTHER EXTREMITY WITH ULCERATION |
| I70.36 | ATHEROSCLEROSIS OF UNSPECIFIED TYPE OF BYPASS GRAFT(S) OF THE EXTREMITIES WITH GANGRENE |
| I70.361 | ATHEROSCLEROSIS OF UNSPECIFIED TYPE OF BYPASS GRAFT(S) OF THE EXTREMITIES WITH GANGRENE, RIGHT LEG |
| I70.362 | ATHEROSCLEROSIS OF UNSPECIFIED TYPE OF BYPASS GRAFT(S) OF THE EXTREMITIES WITH GANGRENE, LEFT LEG |
| I70.363 | ATHEROSCLEROSIS OF UNSPECIFIED TYPE OF BYPASS GRAFT(S) OF THE EXTREMITIES WITH GANGRENE, BILATERAL LEGS |
| I70.368 | ATHEROSCLEROSIS OF UNSPECIFIED TYPE OF BYPASS GRAFT(S) OF THE EXTREMITIES WITH GANGRENE, OTHER EXTREMITY |
| I70.369 | ATHEROSCLEROSIS OF UNSPECIFIED TYPE OF BYPASS GRAFT(S) OF THE EXTREMITIES WITH GANGRENE, UNSPECIFIED EXTREMITY |
| I70.39 | OTHER ATHEROSCLEROSIS OF UNSPECIFIED TYPE OF BYPASS GRAFT(S) OF THE EXTREMITIES |
| I70.391 | OTHER ATHEROSCLEROSIS OF UNSPECIFIED TYPE OF BYPASS GRAFT(S) OF THE EXTREMITIES, RIGHT LEG |
| I70.392 | OTHER ATHEROSCLEROSIS OF UNSPECIFIED TYPE OF BYPASS GRAFT(S) OF THE EXTREMITIES, LEFT LEG |
| I70.393 | OTHER ATHEROSCLEROSIS OF UNSPECIFIED TYPE OF BYPASS GRAFT(S) OF THE EXTREMITIES, BILATERAL LEGS |
| I70.398 | OTHER ATHEROSCLEROSIS OF UNSPECIFIED TYPE OF BYPASS GRAFT(S) OF THE EXTREMITIES, OTHER EXTREMITY |
| I70.399 | OTHER ATHEROSCLEROSIS OF UNSPECIFIED TYPE OF BYPASS GRAFT(S) OF THE EXTREMITIES, UNSPECIFIED EXTREMITY |
| I70.4 | ATHEROSCLEROSIS OF AUTOLOGOUS VEIN BYPASS GRAFT(S) OF THE EXTREMITIES |
| I70.40 | UNSPECIFIED ATHEROSCLEROSIS OF AUTOLOGOUS VEIN BYPASS GRAFT(S) OF THE EXTREMITIES |
| I70.401 | UNSPECIFIED ATHEROSCLEROSIS OF AUTOLOGOUS VEIN BYPASS GRAFT(S) OF THE EXTREMITIES, RIGHT LEG |
| I70.402 | UNSPECIFIED ATHEROSCLEROSIS OF AUTOLOGOUS VEIN BYPASS GRAFT(S) OF THE EXTREMITIES, LEFT LEG |
| I70.403 | UNSPECIFIED ATHEROSCLEROSIS OF AUTOLOGOUS VEIN BYPASS GRAFT(S) OF THE EXTREMITIES, BILATERAL LEGS |
| I70.408 | UNSPECIFIED ATHEROSCLEROSIS OF AUTOLOGOUS VEIN BYPASS GRAFT(S) OF THE EXTREMITIES, OTHER EXTREMITY |
| I70.409 | UNSPECIFIED ATHEROSCLEROSIS OF AUTOLOGOUS VEIN BYPASS GRAFT(S) OF THE EXTREMITIES, UNSPECIFIED EXTREMITY |
| I70.41 | ATHEROSCLEROSIS OF AUTOLOGOUS VEIN BYPASS GRAFT(S) OF THE EXTREMITIES WITH INTERMITTENT CLAUDICATION |
| I70.411 | ATHEROSCLEROSIS OF AUTOLOGOUS VEIN BYPASS GRAFT(S) OF THE EXTREMITIES WITH INTERMITTENT CLAUDICATION, RIGHT LEG |
| I70.412 | ATHEROSCLEROSIS OF AUTOLOGOUS VEIN BYPASS GRAFT(S) OF THE EXTREMITIES WITH INTERMITTENT CLAUDICATION, LEFT LEG |
| I70.413 | ATHEROSCLEROSIS OF AUTOLOGOUS VEIN BYPASS GRAFT(S) OF THE EXTREMITIES WITH INTERMITTENT CLAUDICATION, BILATERAL LEGS |
| I70.418 | ATHEROSCLEROSIS OF AUTOLOGOUS VEIN BYPASS GRAFT(S) OF THE EXTREMITIES WITH INTERMITTENT CLAUDICATION, OTHER EXTREMITY |
| I70.419 | ATHEROSCLEROSIS OF AUTOLOGOUS VEIN BYPASS GRAFT(S) OF THE EXTREMITIES WITH INTERMITTENT CLAUDICATION, UNSPECIFIED EXTREMITY |
| I70.42 | ATHEROSCLEROSIS OF AUTOLOGOUS VEIN BYPASS GRAFT(S) OF THE EXTREMITIES WITH REST PAIN |
| I70.421 | ATHEROSCLEROSIS OF AUTOLOGOUS VEIN BYPASS GRAFT(S) OF THE EXTREMITIES WITH REST PAIN, RIGHT LEG |
| I70.422 | ATHEROSCLEROSIS OF AUTOLOGOUS VEIN BYPASS GRAFT(S) OF THE EXTREMITIES WITH REST PAIN, LEFT LEG |
| I70.423 | ATHEROSCLEROSIS OF AUTOLOGOUS VEIN BYPASS GRAFT(S) OF THE EXTREMITIES WITH REST PAIN, BILATERAL LEGS |
| I70.428 | ATHEROSCLEROSIS OF AUTOLOGOUS VEIN BYPASS GRAFT(S) OF THE EXTREMITIES WITH REST PAIN, OTHER EXTREMITY |
| I70.429 | ATHEROSCLEROSIS OF AUTOLOGOUS VEIN BYPASS GRAFT(S) OF THE EXTREMITIES WITH REST PAIN, UNSPECIFIED EXTREMITY |
| I70.43 | ATHEROSCLEROSIS OF AUTOLOGOUS VEIN BYPASS GRAFT(S) OF THE RIGHT LEG WITH ULCERATION |
| I70.431 | ATHEROSCLEROSIS OF AUTOLOGOUS VEIN BYPASS GRAFT(S) OF THE RIGHT LEG WITH ULCERATION OF THIGH |
| I70.432 | ATHEROSCLEROSIS OF AUTOLOGOUS VEIN BYPASS GRAFT(S) OF THE RIGHT LEG WITH ULCERATION OF CALF |
| I70.433 | ATHEROSCLEROSIS OF AUTOLOGOUS VEIN BYPASS GRAFT(S) OF THE RIGHT LEG WITH ULCERATION OF ANKLE |
| I70.434 | ATHEROSCLEROSIS OF AUTOLOGOUS VEIN BYPASS GRAFT(S) OF THE RIGHT LEG WITH ULCERATION OF HEEL AND MIDFOOT |
| I70.435 | ATHEROSCLEROSIS OF AUTOLOGOUS VEIN BYPASS GRAFT(S) OF THE RIGHT LEG WITH ULCERATION OF OTHER PART OF FOOT |
| I70.438 | ATHEROSCLEROSIS OF AUTOLOGOUS VEIN BYPASS GRAFT(S) OF THE RIGHT LEG WITH ULCERATION OF OTHER PART OF LOWER LEG |
| I70.439 | ATHEROSCLEROSIS OF AUTOLOGOUS VEIN BYPASS GRAFT(S) OF THE RIGHT LEG WITH ULCERATION OF UNSPECIFIED SITE |
| I70.44 | ATHEROSCLEROSIS OF AUTOLOGOUS VEIN BYPASS GRAFT(S) OF THE LEFT LEG WITH ULCERATION |
| I70.441 | ATHEROSCLEROSIS OF AUTOLOGOUS VEIN BYPASS GRAFT(S) OF THE LEFT LEG WITH ULCERATION OF THIGH |
| I70.442 | ATHEROSCLEROSIS OF AUTOLOGOUS VEIN BYPASS GRAFT(S) OF THE LEFT LEG WITH ULCERATION OF CALF |
| I70.443 | ATHEROSCLEROSIS OF AUTOLOGOUS VEIN BYPASS GRAFT(S) OF THE LEFT LEG WITH ULCERATION OF ANKLE |
| I70.444 | ATHEROSCLEROSIS OF AUTOLOGOUS VEIN BYPASS GRAFT(S) OF THE LEFT LEG WITH ULCERATION OF HEEL AND MIDFOOT |
| I70.445 | ATHEROSCLEROSIS OF AUTOLOGOUS VEIN BYPASS GRAFT(S) OF THE LEFT LEG WITH ULCERATION OF OTHER PART OF FOOT |
| I70.448 | ATHEROSCLEROSIS OF AUTOLOGOUS VEIN BYPASS GRAFT(S) OF THE LEFT LEG WITH ULCERATION OF OTHER PART OF LOWER LEG |
| I70.449 | ATHEROSCLEROSIS OF AUTOLOGOUS VEIN BYPASS GRAFT(S) OF THE LEFT LEG WITH ULCERATION OF UNSPECIFIED SITE |
| I70.45 | ATHEROSCLEROSIS OF AUTOLOGOUS VEIN BYPASS GRAFT(S) OF OTHER EXTREMITY WITH ULCERATION |
| I70.46 | ATHEROSCLEROSIS OF AUTOLOGOUS VEIN BYPASS GRAFT(S) OF THE EXTREMITIES WITH GANGRENE |
| I70.461 | ATHEROSCLEROSIS OF AUTOLOGOUS VEIN BYPASS GRAFT(S) OF THE EXTREMITIES WITH GANGRENE, RIGHT LEG |
| I70.462 | ATHEROSCLEROSIS OF AUTOLOGOUS VEIN BYPASS GRAFT(S) OF THE EXTREMITIES WITH GANGRENE, LEFT LEG |
| I70.463 | ATHEROSCLEROSIS OF AUTOLOGOUS VEIN BYPASS GRAFT(S) OF THE EXTREMITIES WITH GANGRENE, BILATERAL LEGS |
| I70.468 | ATHEROSCLEROSIS OF AUTOLOGOUS VEIN BYPASS GRAFT(S) OF THE EXTREMITIES WITH GANGRENE, OTHER EXTREMITY |
| I70.469 | ATHEROSCLEROSIS OF AUTOLOGOUS VEIN BYPASS GRAFT(S) OF THE EXTREMITIES WITH GANGRENE, UNSPECIFIED EXTREMITY |
| I70.49 | OTHER ATHEROSCLEROSIS OF AUTOLOGOUS VEIN BYPASS GRAFT(S) OF THE EXTREMITIES |
| I70.491 | OTHER ATHEROSCLEROSIS OF AUTOLOGOUS VEIN BYPASS GRAFT(S) OF THE EXTREMITIES, RIGHT LEG |
| I70.492 | OTHER ATHEROSCLEROSIS OF AUTOLOGOUS VEIN BYPASS GRAFT(S) OF THE EXTREMITIES, LEFT LEG |
| I70.493 | OTHER ATHEROSCLEROSIS OF AUTOLOGOUS VEIN BYPASS GRAFT(S) OF THE EXTREMITIES, BILATERAL LEGS |
| I70.498 | OTHER ATHEROSCLEROSIS OF AUTOLOGOUS VEIN BYPASS GRAFT(S) OF THE EXTREMITIES, OTHER EXTREMITY |
| I70.499 | OTHER ATHEROSCLEROSIS OF AUTOLOGOUS VEIN BYPASS GRAFT(S) OF THE EXTREMITIES, UNSPECIFIED EXTREMITY |
| I70.5 | ATHEROSCLEROSIS OF NONAUTOLOGOUS BIOLOGICAL BYPASS GRAFT(S) OF THE EXTREMITIES |
| I70.50 | UNSPECIFIED ATHEROSCLEROSIS OF NONAUTOLOGOUS BIOLOGICAL BYPASS GRAFT(S) OF THE EXTREMITIES |
| I70.501 | UNSPECIFIED ATHEROSCLEROSIS OF NONAUTOLOGOUS BIOLOGICAL BYPASS GRAFT(S) OF THE EXTREMITIES, RIGHT LEG |
| I70.502 | UNSPECIFIED ATHEROSCLEROSIS OF NONAUTOLOGOUS BIOLOGICAL BYPASS GRAFT(S) OF THE EXTREMITIES, LEFT LEG |
| I70.503 | UNSPECIFIED ATHEROSCLEROSIS OF NONAUTOLOGOUS BIOLOGICAL BYPASS GRAFT(S) OF THE EXTREMITIES, BILATERAL LEGS |
| I70.508 | UNSPECIFIED ATHEROSCLEROSIS OF NONAUTOLOGOUS BIOLOGICAL BYPASS GRAFT(S) OF THE EXTREMITIES, OTHER EXTREMITY |
| I70.509 | UNSPECIFIED ATHEROSCLEROSIS OF NONAUTOLOGOUS BIOLOGICAL BYPASS GRAFT(S) OF THE EXTREMITIES, UNSPECIFIED EXTREMITY |
| I70.51 | ATHEROSCLEROSIS OF NONAUTOLOGOUS BIOLOGICAL BYPASS GRAFT(S) OF THE EXTREMITIES INTERMITTENT CLAUDICATION |
| I70.511 | ATHEROSCLEROSIS OF NONAUTOLOGOUS BIOLOGICAL BYPASS GRAFT(S) OF THE EXTREMITIES WITH INTERMITTENT CLAUDICATION, RIGHT LEG |
| I70.512 | ATHEROSCLEROSIS OF NONAUTOLOGOUS BIOLOGICAL BYPASS GRAFT(S) OF THE EXTREMITIES WITH INTERMITTENT CLAUDICATION, LEFT LEG |
| I70.513 | ATHEROSCLEROSIS OF NONAUTOLOGOUS BIOLOGICAL BYPASS GRAFT(S) OF THE EXTREMITIES WITH INTERMITTENT CLAUDICATION, BILATERAL LEGS |
| I70.518 | ATHEROSCLEROSIS OF NONAUTOLOGOUS BIOLOGICAL BYPASS GRAFT(S) OF THE EXTREMITIES WITH INTERMITTENT CLAUDICATION, OTHER EXTREMITY |
| I70.519 | ATHEROSCLEROSIS OF NONAUTOLOGOUS BIOLOGICAL BYPASS GRAFT(S) OF THE EXTREMITIES WITH INTERMITTENT CLAUDICATION, UNSPECIFIED EXTREMITY |
| I70.52 | ATHEROSCLEROSIS OF NONAUTOLOGOUS BIOLOGICAL BYPASS GRAFT(S) OF THE EXTREMITIES WITH REST PAIN |
| I70.521 | ATHEROSCLEROSIS OF NONAUTOLOGOUS BIOLOGICAL BYPASS GRAFT(S) OF THE EXTREMITIES WITH REST PAIN, RIGHT LEG |
| I70.522 | ATHEROSCLEROSIS OF NONAUTOLOGOUS BIOLOGICAL BYPASS GRAFT(S) OF THE EXTREMITIES WITH REST PAIN, LEFT LEG |
| I70.523 | ATHEROSCLEROSIS OF NONAUTOLOGOUS BIOLOGICAL BYPASS GRAFT(S) OF THE EXTREMITIES WITH REST PAIN, BILATERAL LEGS |
| I70.528 | ATHEROSCLEROSIS OF NONAUTOLOGOUS BIOLOGICAL BYPASS GRAFT(S) OF THE EXTREMITIES WITH REST PAIN, OTHER EXTREMITY |
| I70.529 | ATHEROSCLEROSIS OF NONAUTOLOGOUS BIOLOGICAL BYPASS GRAFT(S) OF THE EXTREMITIES WITH REST PAIN, UNSPECIFIED EXTREMITY |
| I70.53 | ATHEROSCLEROSIS OF NONAUTOLOGOUS BIOLOGICAL BYPASS GRAFT(S) OF THE RIGHT LEG WITH ULCERATION |
| I70.531 | ATHEROSCLEROSIS OF NONAUTOLOGOUS BIOLOGICAL BYPASS GRAFT(S) OF THE RIGHT LEG WITH ULCERATION OF THIGH |
| I70.532 | ATHEROSCLEROSIS OF NONAUTOLOGOUS BIOLOGICAL BYPASS GRAFT(S) OF THE RIGHT LEG WITH ULCERATION OF CALF |
| I70.533 | ATHEROSCLEROSIS OF NONAUTOLOGOUS BIOLOGICAL BYPASS GRAFT(S) OF THE RIGHT LEG WITH ULCERATION OF ANKLE |
| I70.534 | ATHEROSCLEROSIS OF NONAUTOLOGOUS BIOLOGICAL BYPASS GRAFT(S) OF THE RIGHT LEG WITH ULCERATION OF HEEL AND MIDFOOT |
| I70.535 | ATHEROSCLEROSIS OF NONAUTOLOGOUS BIOLOGICAL BYPASS GRAFT(S) OF THE RIGHT LEG WITH ULCERATION OF OTHER PART OF FOOT |
| I70.538 | ATHEROSCLEROSIS OF NONAUTOLOGOUS BIOLOGICAL BYPASS GRAFT(S) OF THE RIGHT LEG WITH ULCERATION OF OTHER PART OF LOWER LEG |
| I70.539 | ATHEROSCLEROSIS OF NONAUTOLOGOUS BIOLOGICAL BYPASS GRAFT(S) OF THE RIGHT LEG WITH ULCERATION OF UNSPECIFIED SITE |
| I70.54 | ATHEROSCLEROSIS OF NONAUTOLOGOUS BIOLOGICAL BYPASS GRAFT(S) OF THE LEFT LEG WITH ULCERATION |
| I70.541 | ATHEROSCLEROSIS OF NONAUTOLOGOUS BIOLOGICAL BYPASS GRAFT(S) OF THE LEFT LEG WITH ULCERATION OF THIGH |
| I70.542 | ATHEROSCLEROSIS OF NONAUTOLOGOUS BIOLOGICAL BYPASS GRAFT(S) OF THE LEFT LEG WITH ULCERATION OF CALF |
| I70.543 | ATHEROSCLEROSIS OF NONAUTOLOGOUS BIOLOGICAL BYPASS GRAFT(S) OF THE LEFT LEG WITH ULCERATION OF ANKLE |
| I70.544 | ATHEROSCLEROSIS OF NONAUTOLOGOUS BIOLOGICAL BYPASS GRAFT(S) OF THE LEFT LEG WITH ULCERATION OF HEEL AND MIDFOOT |
| I70.545 | ATHEROSCLEROSIS OF NONAUTOLOGOUS BIOLOGICAL BYPASS GRAFT(S) OF THE LEFT LEG WITH ULCERATION OF OTHER PART OF FOOT |
| I70.548 | ATHEROSCLEROSIS OF NONAUTOLOGOUS BIOLOGICAL BYPASS GRAFT(S) OF THE LEFT LEG WITH ULCERATION OF OTHER PART OF LOWER LEG |
| I70.549 | ATHEROSCLEROSIS OF NONAUTOLOGOUS BIOLOGICAL BYPASS GRAFT(S) OF THE LEFT LEG WITH ULCERATION OF UNSPECIFIED SITE |
| I70.55 | ATHEROSCLEROSIS OF NONAUTOLOGOUS BIOLOGICAL BYPASS GRAFT(S) OF OTHER EXTREMITY WITH ULCERATION |
| I70.56 | ATHEROSCLEROSIS OF NONAUTOLOGOUS BIOLOGICAL BYPASS GRAFT(S) OF THE EXTREMITIES WITH GANGRENE |
| I70.561 | ATHEROSCLEROSIS OF NONAUTOLOGOUS BIOLOGICAL BYPASS GRAFT(S) OF THE EXTREMITIES WITH GANGRENE, RIGHT LEG |
| I70.562 | ATHEROSCLEROSIS OF NONAUTOLOGOUS BIOLOGICAL BYPASS GRAFT(S) OF THE EXTREMITIES WITH GANGRENE, LEFT LEG |
| I70.563 | ATHEROSCLEROSIS OF NONAUTOLOGOUS BIOLOGICAL BYPASS GRAFT(S) OF THE EXTREMITIES WITH GANGRENE, BILATERAL LEGS |
| I70.568 | ATHEROSCLEROSIS OF NONAUTOLOGOUS BIOLOGICAL BYPASS GRAFT(S) OF THE EXTREMITIES WITH GANGRENE, OTHER EXTREMITY |
| I70.569 | ATHEROSCLEROSIS OF NONAUTOLOGOUS BIOLOGICAL BYPASS GRAFT(S) OF THE EXTREMITIES WITH GANGRENE, UNSPECIFIED EXTREMITY |
| I70.59 | OTHER ATHEROSCLEROSIS OF NONAUTOLOGOUS BIOLOGICAL BYPASS GRAFT(S) OF THE EXTREMITIES |
| I70.591 | OTHER ATHEROSCLEROSIS OF NONAUTOLOGOUS BIOLOGICAL BYPASS GRAFT(S) OF THE EXTREMITIES, RIGHT LEG |
| I70.592 | OTHER ATHEROSCLEROSIS OF NONAUTOLOGOUS BIOLOGICAL BYPASS GRAFT(S) OF THE EXTREMITIES, LEFT LEG |
| I70.593 | OTHER ATHEROSCLEROSIS OF NONAUTOLOGOUS BIOLOGICAL BYPASS GRAFT(S) OF THE EXTREMITIES, BILATERAL LEGS |
| I70.598 | OTHER ATHEROSCLEROSIS OF NONAUTOLOGOUS BIOLOGICAL BYPASS GRAFT(S) OF THE EXTREMITIES, OTHER EXTREMITY |
| I70.599 | OTHER ATHEROSCLEROSIS OF NONAUTOLOGOUS BIOLOGICAL BYPASS GRAFT(S) OF THE EXTREMITIES, UNSPECIFIED EXTREMITY |
| I70.6 | ATHEROSCLEROSIS OF NONBIOLOGICAL BYPASS GRAFT(S) OF THE EXTREMITIES |
| I70.60 | UNSPECIFIED ATHEROSCLEROSIS OF NONBIOLOGICAL BYPASS GRAFT(S) OF THE EXTREMITIES |
| I70.601 | UNSPECIFIED ATHEROSCLEROSIS OF NONBIOLOGICAL BYPASS GRAFT(S) OF THE EXTREMITIES, RIGHT LEG |
| I70.602 | UNSPECIFIED ATHEROSCLEROSIS OF NONBIOLOGICAL BYPASS GRAFT(S) OF THE EXTREMITIES, LEFT LEG |
| I70.603 | UNSPECIFIED ATHEROSCLEROSIS OF NONBIOLOGICAL BYPASS GRAFT(S) OF THE EXTREMITIES, BILATERAL LEGS |
| I70.608 | UNSPECIFIED ATHEROSCLEROSIS OF NONBIOLOGICAL BYPASS GRAFT(S) OF THE EXTREMITIES, OTHER EXTREMITY |
| I70.609 | UNSPECIFIED ATHEROSCLEROSIS OF NONBIOLOGICAL BYPASS GRAFT(S) OF THE EXTREMITIES, UNSPECIFIED EXTREMITY |
| I70.61 | ATHEROSCLEROSIS OF NONBIOLOGICAL BYPASS GRAFT(S) OF THE EXTREMITIES WITH INTERMITTENT CLAUDICATION |
| I70.611 | ATHEROSCLEROSIS OF NONBIOLOGICAL BYPASS GRAFT(S) OF THE EXTREMITIES WITH INTERMITTENT CLAUDICATION, RIGHT LEG |
| I70.612 | ATHEROSCLEROSIS OF NONBIOLOGICAL BYPASS GRAFT(S) OF THE EXTREMITIES WITH INTERMITTENT CLAUDICATION, LEFT LEG |
| I70.613 | ATHEROSCLEROSIS OF NONBIOLOGICAL BYPASS GRAFT(S) OF THE EXTREMITIES WITH INTERMITTENT CLAUDICATION, BILATERAL LEGS |
| I70.618 | ATHEROSCLEROSIS OF NONBIOLOGICAL BYPASS GRAFT(S) OF THE EXTREMITIES WITH INTERMITTENT CLAUDICATION, OTHER EXTREMITY |
| I70.619 | ATHEROSCLEROSIS OF NONBIOLOGICAL BYPASS GRAFT(S) OF THE EXTREMITIES WITH INTERMITTENT CLAUDICATION, UNSPECIFIED EXTREMITY |
| I70.62 | ATHEROSCLEROSIS OF NONBIOLOGICAL BYPASS GRAFT(S) OF THE EXTREMITIES WITH REST PAIN |
| I70.621 | ATHEROSCLEROSIS OF NONBIOLOGICAL BYPASS GRAFT(S) OF THE EXTREMITIES WITH REST PAIN, RIGHT LEG |
| I70.622 | ATHEROSCLEROSIS OF NONBIOLOGICAL BYPASS GRAFT(S) OF THE EXTREMITIES WITH REST PAIN, LEFT LEG |
| I70.623 | ATHEROSCLEROSIS OF NONBIOLOGICAL BYPASS GRAFT(S) OF THE EXTREMITIES WITH REST PAIN, BILATERAL LEGS |
| I70.628 | ATHEROSCLEROSIS OF NONBIOLOGICAL BYPASS GRAFT(S) OF THE EXTREMITIES WITH REST PAIN, OTHER EXTREMITY |
| I70.629 | ATHEROSCLEROSIS OF NONBIOLOGICAL BYPASS GRAFT(S) OF THE EXTREMITIES WITH REST PAIN, UNSPECIFIED EXTREMITY |
| I70.63 | ATHEROSCLEROSIS OF NONBIOLOGICAL BYPASS GRAFT(S) OF THE RIGHT LEG WITH ULCERATION |
| I70.631 | ATHEROSCLEROSIS OF NONBIOLOGICAL BYPASS GRAFT(S) OF THE RIGHT LEG WITH ULCERATION OF THIGH |
| I70.632 | ATHEROSCLEROSIS OF NONBIOLOGICAL BYPASS GRAFT(S) OF THE RIGHT LEG WITH ULCERATION OF CALF |
| I70.633 | ATHEROSCLEROSIS OF NONBIOLOGICAL BYPASS GRAFT(S) OF THE RIGHT LEG WITH ULCERATION OF ANKLE |
| I70.634 | ATHEROSCLEROSIS OF NONBIOLOGICAL BYPASS GRAFT(S) OF THE RIGHT LEG WITH ULCERATION OF HEEL AND MIDFOOT |
| I70.635 | ATHEROSCLEROSIS OF NONBIOLOGICAL BYPASS GRAFT(S) OF THE RIGHT LEG WITH ULCERATION OF OTHER PART OF FOOT |
| I70.638 | ATHEROSCLEROSIS OF NONBIOLOGICAL BYPASS GRAFT(S) OF THE RIGHT LEG WITH ULCERATION OF OTHER PART OF LOWER LEG |
| I70.639 | ATHEROSCLEROSIS OF NONBIOLOGICAL BYPASS GRAFT(S) OF THE RIGHT LEG WITH ULCERATION OF UNSPECIFIED SITE |
| I70.64 | ATHEROSCLEROSIS OF NONBIOLOGICAL BYPASS GRAFT(S) OF THE LEFT LEG WITH ULCERATION |
| I70.641 | ATHEROSCLEROSIS OF NONBIOLOGICAL BYPASS GRAFT(S) OF THE LEFT LEG WITH ULCERATION OF THIGH |
| I70.642 | ATHEROSCLEROSIS OF NONBIOLOGICAL BYPASS GRAFT(S) OF THE LEFT LEG WITH ULCERATION OF CALF |
| I70.643 | ATHEROSCLEROSIS OF NONBIOLOGICAL BYPASS GRAFT(S) OF THE LEFT LEG WITH ULCERATION OF ANKLE |
| I70.644 | ATHEROSCLEROSIS OF NONBIOLOGICAL BYPASS GRAFT(S) OF THE LEFT LEG WITH ULCERATION OF HEEL AND MIDFOOT |
| I70.645 | ATHEROSCLEROSIS OF NONBIOLOGICAL BYPASS GRAFT(S) OF THE LEFT LEG WITH ULCERATION OF OTHER PART OF FOOT |
| I70.648 | ATHEROSCLEROSIS OF NONBIOLOGICAL BYPASS GRAFT(S) OF THE LEFT LEG WITH ULCERATION OF OTHER PART OF LOWER LEG |
| I70.649 | ATHEROSCLEROSIS OF NONBIOLOGICAL BYPASS GRAFT(S) OF THE LEFT LEG WITH ULCERATION OF UNSPECIFIED SITE |
| I70.65 | ATHEROSCLEROSIS OF NONBIOLOGICAL BYPASS GRAFT(S) OF OTHER EXTREMITY WITH ULCERATION |
| I70.66 | ATHEROSCLEROSIS OF NONBIOLOGICAL BYPASS GRAFT(S) OF THE EXTREMITIES WITH GANGRENE |
| I70.661 | ATHEROSCLEROSIS OF NONBIOLOGICAL BYPASS GRAFT(S) OF THE EXTREMITIES WITH GANGRENE, RIGHT LEG |
| I70.662 | ATHEROSCLEROSIS OF NONBIOLOGICAL BYPASS GRAFT(S) OF THE EXTREMITIES WITH GANGRENE, LEFT LEG |
| I70.663 | ATHEROSCLEROSIS OF NONBIOLOGICAL BYPASS GRAFT(S) OF THE EXTREMITIES WITH GANGRENE, BILATERAL LEGS |
| I70.668 | ATHEROSCLEROSIS OF NONBIOLOGICAL BYPASS GRAFT(S) OF THE EXTREMITIES WITH GANGRENE, OTHER EXTREMITY |
| I70.669 | ATHEROSCLEROSIS OF NONBIOLOGICAL BYPASS GRAFT(S) OF THE EXTREMITIES WITH GANGRENE, UNSPECIFIED EXTREMITY |
| I70.69 | OTHER ATHEROSCLEROSIS OF NONBIOLOGICAL BYPASS GRAFT(S) OF THE EXTREMITIES |
| I70.691 | OTHER ATHEROSCLEROSIS OF NONBIOLOGICAL BYPASS GRAFT(S) OF THE EXTREMITIES, RIGHT LEG |
| I70.692 | OTHER ATHEROSCLEROSIS OF NONBIOLOGICAL BYPASS GRAFT(S) OF THE EXTREMITIES, LEFT LEG |
| I70.693 | OTHER ATHEROSCLEROSIS OF NONBIOLOGICAL BYPASS GRAFT(S) OF THE EXTREMITIES, BILATERAL LEGS |
| I70.698 | OTHER ATHEROSCLEROSIS OF NONBIOLOGICAL BYPASS GRAFT(S) OF THE EXTREMITIES, OTHER EXTREMITY |
| I70.699 | OTHER ATHEROSCLEROSIS OF NONBIOLOGICAL BYPASS GRAFT(S) OF THE EXTREMITIES, UNSPECIFIED EXTREMITY |
| I70.7 | ATHEROSCLEROSIS OF OTHER TYPE OF BYPASS GRAFT(S) OF THE EXTREMITIES |
| I70.70 | UNSPECIFIED ATHEROSCLEROSIS OF OTHER TYPE OF BYPASS GRAFT(S) OF THE EXTREMITIES |
| I70.701 | UNSPECIFIED ATHEROSCLEROSIS OF OTHER TYPE OF BYPASS GRAFT(S) OF THE EXTREMITIES, RIGHT LEG |
| I70.702 | UNSPECIFIED ATHEROSCLEROSIS OF OTHER TYPE OF BYPASS GRAFT(S) OF THE EXTREMITIES, LEFT LEG |
| I70.703 | UNSPECIFIED ATHEROSCLEROSIS OF OTHER TYPE OF BYPASS GRAFT(S) OF THE EXTREMITIES, BILATERAL LEGS |
| I70.708 | UNSPECIFIED ATHEROSCLEROSIS OF OTHER TYPE OF BYPASS GRAFT(S) OF THE EXTREMITIES, OTHER EXTREMITY |
| I70.709 | UNSPECIFIED ATHEROSCLEROSIS OF OTHER TYPE OF BYPASS GRAFT(S) OF THE EXTREMITIES, UNSPECIFIED EXTREMITY |
| I70.71 | ATHEROSCLEROSIS OF OTHER TYPE OF BYPASS GRAFT(S) OF THE EXTREMITIES WITH INTERMITTENT CLAUDICATION |
| I70.711 | ATHEROSCLEROSIS OF OTHER TYPE OF BYPASS GRAFT(S) OF THE EXTREMITIES WITH INTERMITTENT CLAUDICATION, RIGHT LEG |
| I70.712 | ATHEROSCLEROSIS OF OTHER TYPE OF BYPASS GRAFT(S) OF THE EXTREMITIES WITH INTERMITTENT CLAUDICATION, LEFT LEG |
| I70.713 | ATHEROSCLEROSIS OF OTHER TYPE OF BYPASS GRAFT(S) OF THE EXTREMITIES WITH INTERMITTENT CLAUDICATION, BILATERAL LEGS |
| I70.718 | ATHEROSCLEROSIS OF OTHER TYPE OF BYPASS GRAFT(S) OF THE EXTREMITIES WITH INTERMITTENT CLAUDICATION, OTHER EXTREMITY |
| I70.719 | ATHEROSCLEROSIS OF OTHER TYPE OF BYPASS GRAFT(S) OF THE EXTREMITIES WITH INTERMITTENT CLAUDICATION, UNSPECIFIED EXTREMITY |
| I70.72 | ATHEROSCLEROSIS OF OTHER TYPE OF BYPASS GRAFT(S) OF THE EXTREMITIES WITH REST PAIN |
| I70.721 | ATHEROSCLEROSIS OF OTHER TYPE OF BYPASS GRAFT(S) OF THE EXTREMITIES WITH REST PAIN, RIGHT LEG |
| I70.722 | ATHEROSCLEROSIS OF OTHER TYPE OF BYPASS GRAFT(S) OF THE EXTREMITIES WITH REST PAIN, LEFT LEG |
| I70.723 | ATHEROSCLEROSIS OF OTHER TYPE OF BYPASS GRAFT(S) OF THE EXTREMITIES WITH REST PAIN, BILATERAL LEGS |
| I70.728 | ATHEROSCLEROSIS OF OTHER TYPE OF BYPASS GRAFT(S) OF THE EXTREMITIES WITH REST PAIN, OTHER EXTREMITY |
| I70.729 | ATHEROSCLEROSIS OF OTHER TYPE OF BYPASS GRAFT(S) OF THE EXTREMITIES WITH REST PAIN, UNSPECIFIED EXTREMITY |
| I70.73 | ATHEROSCLEROSIS OF OTHER TYPE OF BYPASS GRAFT(S) OF THE RIGHT LEG WITH ULCERATION |
| I70.731 | ATHEROSCLEROSIS OF OTHER TYPE OF BYPASS GRAFT(S) OF THE RIGHT LEG WITH ULCERATION OF THIGH |
| I70.732 | ATHEROSCLEROSIS OF OTHER TYPE OF BYPASS GRAFT(S) OF THE RIGHT LEG WITH ULCERATION OF CALF |
| I70.733 | ATHEROSCLEROSIS OF OTHER TYPE OF BYPASS GRAFT(S) OF THE RIGHT LEG WITH ULCERATION OF ANKLE |
| I70.734 | ATHEROSCLEROSIS OF OTHER TYPE OF BYPASS GRAFT(S) OF THE RIGHT LEG WITH ULCERATION OF HEEL AND MIDFOOT |
| I70.735 | ATHEROSCLEROSIS OF OTHER TYPE OF BYPASS GRAFT(S) OF THE RIGHT LEG WITH ULCERATION OF OTHER PART OF FOOT |
| I70.738 | ATHEROSCLEROSIS OF OTHER TYPE OF BYPASS GRAFT(S) OF THE RIGHT LEG WITH ULCERATION OF OTHER PART OF LOWER LEG |
| I70.739 | ATHEROSCLEROSIS OF OTHER TYPE OF BYPASS GRAFT(S) OF THE RIGHT LEG WITH ULCERATION OF UNSPECIFIED SITE |
| I70.74 | ATHEROSCLEROSIS OF OTHER TYPE OF BYPASS GRAFT(S) OF THE LEFT LEG WITH ULCERATION |
| I70.741 | ATHEROSCLEROSIS OF OTHER TYPE OF BYPASS GRAFT(S) OF THE LEFT LEG WITH ULCERATION OF THIGH |
| I70.742 | ATHEROSCLEROSIS OF OTHER TYPE OF BYPASS GRAFT(S) OF THE LEFT LEG WITH ULCERATION OF CALF |
| I70.743 | ATHEROSCLEROSIS OF OTHER TYPE OF BYPASS GRAFT(S) OF THE LEFT LEG WITH ULCERATION OF ANKLE |
| I70.744 | ATHEROSCLEROSIS OF OTHER TYPE OF BYPASS GRAFT(S) OF THE LEFT LEG WITH ULCERATION OF HEEL AND MIDFOOT |
| I70.745 | ATHEROSCLEROSIS OF OTHER TYPE OF BYPASS GRAFT(S) OF THE LEFT LEG WITH ULCERATION OF OTHER PART OF FOOT |
| I70.748 | ATHEROSCLEROSIS OF OTHER TYPE OF BYPASS GRAFT(S) OF THE LEFT LEG WITH ULCERATION OF OTHER PART OF LOWER LEG |
| I70.749 | ATHEROSCLEROSIS OF OTHER TYPE OF BYPASS GRAFT(S) OF THE LEFT LEG WITH ULCERATION OF UNSPECIFIED SITE |
| I70.75 | ATHEROSCLEROSIS OF OTHER TYPE OF BYPASS GRAFT(S) OF OTHER EXTREMITY WITH ULCERATION |
| I70.76 | ATHEROSCLEROSIS OF OTHER TYPE OF BYPASS GRAFT(S) OF THE EXTREMITIES WITH GANGRENE |
| I70.761 | ATHEROSCLEROSIS OF OTHER TYPE OF BYPASS GRAFT(S) OF THE EXTREMITIES WITH GANGRENE, RIGHT LEG |
| I70.762 | ATHEROSCLEROSIS OF OTHER TYPE OF BYPASS GRAFT(S) OF THE EXTREMITIES WITH GANGRENE, LEFT LEG |
| I70.763 | ATHEROSCLEROSIS OF OTHER TYPE OF BYPASS GRAFT(S) OF THE EXTREMITIES WITH GANGRENE, BILATERAL LEGS |
| I70.768 | ATHEROSCLEROSIS OF OTHER TYPE OF BYPASS GRAFT(S) OF THE EXTREMITIES WITH GANGRENE, OTHER EXTREMITY |
| I70.769 | ATHEROSCLEROSIS OF OTHER TYPE OF BYPASS GRAFT(S) OF THE EXTREMITIES WITH GANGRENE, UNSPECIFIED EXTREMITY |
| I70.79 | OTHER ATHEROSCLEROSIS OF OTHER TYPE OF BYPASS GRAFT(S) OF THE EXTREMITIES |
| I70.791 | OTHER ATHEROSCLEROSIS OF OTHER TYPE OF BYPASS GRAFT(S) OF THE EXTREMITIES, RIGHT LEG |
| I70.792 | OTHER ATHEROSCLEROSIS OF OTHER TYPE OF BYPASS GRAFT(S) OF THE EXTREMITIES, LEFT LEG |
| I70.793 | OTHER ATHEROSCLEROSIS OF OTHER TYPE OF BYPASS GRAFT(S) OF THE EXTREMITIES, BILATERAL LEGS |
| I70.798 | OTHER ATHEROSCLEROSIS OF OTHER TYPE OF BYPASS GRAFT(S) OF THE EXTREMITIES, OTHER EXTREMITY |
| I70.799 | OTHER ATHEROSCLEROSIS OF OTHER TYPE OF BYPASS GRAFT(S) OF THE EXTREMITIES, UNSPECIFIED EXTREMITY |
| I70.92 | CHRONIC TOTAL OCCLUSION OF ARTERY OF THE EXTREMITIES |
| I73.9 | PERIPHERAL VASCULAR DISEASE, UNSPECIFIED |
| I75.02 | ATHEROEMBOLISM OF LOWER EXTREMITY |
| I75.021 | ATHEROEMBOLISM OF RIGHT LOWER EXTREMITY |
| I75.022 | ATHEROEMBOLISM OF LEFT LOWER EXTREMITY |
| I75.023 | ATHEROEMBOLISM OF BILATERAL LOWER EXTREMITIES |
| I75.029 | ATHEROEMBOLISM OF UNSPECIFIED LOWER EXTREMITY |
